# Neuronal activity-dependent mechanisms of small cell lung cancer pathogenesis

**DOI:** 10.1038/s41586-025-09492-z

**Published:** 2025-09-10

**Authors:** Solomiia Savchuk, Kaylee M. Gentry, Wengang Wang, Elana Carleton, Carlos A. O. Biagi-Junior, Karan Luthria, Belgin Yalçın, Lijun Ni, Hannah C. Farnsworth, Rachel A. Davis, Richard Drexler, Johannes C. Melms, Yin Liu, Lehi Acosta-Alvarez, Griffin G. Hartmann, Elisa C. Pavarino, Jenna LaBelle, Pamelyn J. Woo, Angus M. Toland, Fangfei Qu, Yoon Seok Kim, Mariella G. Filbin, Mark A. Krasnow, Keith L. Ligon, Benjamin Izar, Julien Sage, Bernardo L. Sabatini, Michelle Monje, Humsa S. Venkatesh

**Affiliations:** 1 Department of Neurology and Neurological Sciences, Stanford University, Stanford, CA USA; 2 Department of Neurology, Brigham and Women’s Hospital, Boston, MA USA; 3 Department of Neurobiology, Harvard Medical School, Boston, MA USA; 4 Department of Pediatric Oncology, Dana-Farber Boston Children’s Cancer and Blood Disorders Center, Boston, MA USA; 5 Department of Medicine, Division of Hematology/Oncology, Herbert Irving Comprehensive Cancer Center, Columbia University Irving Medical Center, New York, NY USA; 6 Department of Biochemistry, Stanford University, Stanford, CA USA; 7 Departments of Pediatrics and Genetics, Stanford University, Stanford, CA USA; 8 Department of Pathology, Stanford University, Stanford, CA USA; 9 Howard Hughes Medical Institute, Stanford University, Stanford, CA USA; 10 Department of Pathology, Dana-Farber Cancer Institute, Boston, MA USA; 11 Howard Hughes Medical Institute, Harvard Medical School, Boston, MA USA; 12 Department of Neurology, Harvard Medical School, Boston, MA USA

**Keywords:** Cancer microenvironment, Diseases of the nervous system, Small-cell lung cancer

## Abstract

Neural activity is increasingly recognized as a crucial regulator of cancer growth. In the brain, neuronal activity robustly influences glioma growth through paracrine mechanisms^1^ and by electrochemical integration of malignant cells into neural circuitry via neuron-to-glioma synapses^2,3^. Outside of the central nervous system, innervation of tumours such as prostate, head and neck, breast, pancreatic, and gastrointestinal cancers by peripheral nerves similarly regulates cancer progression^4–12^. However, the extent to which the nervous system regulates small cell lung cancer (SCLC) progression, either in the lung or when growing within the brain, is less well understood. SCLC is a lethal high-grade neuroendocrine tumour that exhibits a strong propensity to metastasize to the brain. Here we demonstrate that in the lung, vagus nerve transection markedly inhibits primary lung tumour development and progression, highlighting a critical role for innervation in SCLC growth. In the brain, SCLC cells co-opt neuronal activity-regulated mechanisms to stimulate growth and progression. Glutamatergic and GABAergic (γ-aminobutyric acid-producing) cortical neuronal activity each drive proliferation of SCLC in the brain through paracrine and synaptic neuron–cancer interactions. SCLC cells form bona fide neuron-to-SCLC synapses and exhibit depolarizing currents with consequent calcium transients in response to neuronal activity; such SCLC cell membrane depolarization is sufficient to promote the growth of intracranial tumours. Together, these findings illustrate that neuronal activity has a crucial role in dictating SCLC pathogenesis.

## Abstract (web summary)

Glutamatergic and GABAergic (γ-aminobutyric acid-producing) cortical neuronal activity drives proliferation of small lung cell cancer via paracrine interactions and through synapses formed with tumour cells.

## Main

The nervous system is emerging as a critical component of the tumour microenvironment that regulates cancer pathobiology. Primary brain cancers such as gliomas exhibit a profound dependency on these neuronal mechanisms through activity-dependent paracrine signalling pathways^1,13–15^ and direct functional integration of malignant glioma cells into electrically active neural circuits^2,3,16^. Although less is known about the role of neurons in brain metastases, breast cancer cells that are metastatic to brain were recently found to occupy the perisynaptic space to usurp glutamate for their growth^17^. Increasing evidence also implicates the nervous system in the regulation of many cancers outside of the central nervous system, including prostate, gastric, colon, head and neck, pancreatic, breast and skin cancers^4–12^. These studies support the emerging principle that the nervous system can profoundly influence cancer pathobiology and highlight the large number of cancers that remain to be examined from this perspective.

SCLC is a lethal high-grade neuroendocrine tumour that accounts for around 15% of all lung cancers, causes more than 200,000 deaths worldwide annually^18^, and has a 60% chance of metastasis by the time of diagnosis, with a particular propensity to metastasize to the brain^19,20^. Experimental mouse models indicate that SCLC can originate from pulmonary neuroendocrine cells^21–23^, a lung epithelial cell type that resides in close proximity to nerve fibres and expresses neurotransmitter receptors^24–26^. SCLC cells exhibit gene expression programmes that resemble those in neurons^27–29^. Higher levels of these neuronal markers correlate with shorter survival and more metastatic disease^30–32^. Recent studies have found that cell-intrinsic neurotransmitter-mediated signalling within the lung promotes SCLC progression^33–35^. Further, neuronal gene expression programmes in SCLC are implicated in driving metastatic progression by facilitating interactions with astrocytes in the brain microenvironment^36^. However, whether neuronal activity is a regulator of SCLC growth within the lung or the brain is not yet understood. We hypothesized that innervation—particularly by the vagus nerve—may influence disease growth in the lung and that nervous system–SCLC interactions may drive tumour progression. Here we investigate the role of neurons and neuronal activity in the progression of primary SCLC in the lung and SCLC in the brain.

## Vagal innervation in primary lung tumours

**Extended Data Fig. 1:**
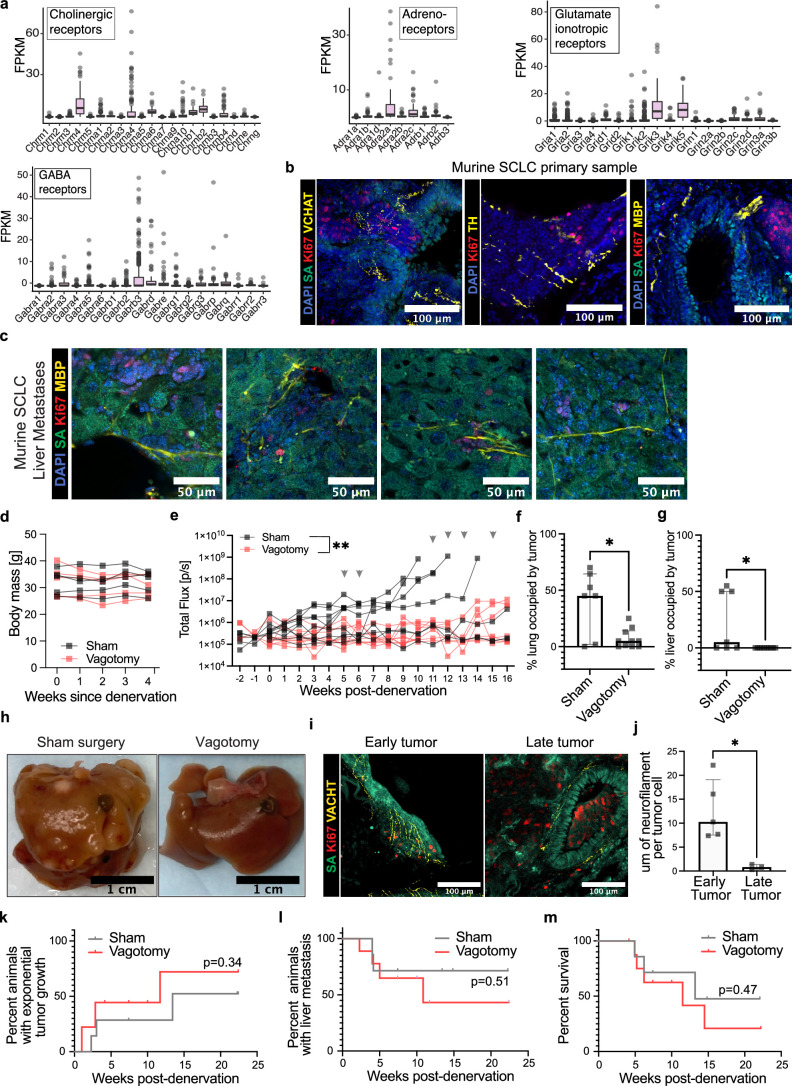
Denervation in genetic model of spontaneously forming SCLC. **a**, Quantification of neurotransmitter receptor gene expression in human samples of primary SCLC (n = 81, for gene list see Supplementary Table 1). **b**, Visualization of tumor innervation in primary lung tumor samples taken from the genetic mouse model of spontaneously forming SCLC. Streptavidin (SA, green) used to mark airways^79^; proliferative cells are labeled with Ki67 (red) to help identify tumors. Vesicular acetylcholine transporter (VAChT), tyrosine hydroxylase (TH), and myelin basic protein (MBP) (yellow) are used to visualize parasympathetic, sympathetic, and myelinated sensory populations of nerve fibers in left, middle and right image, respectively. Scale bar = 100 µm. **c**, Visualization of tumor innervation of liver metastasis in murine RPR2-luc model of SCLC. Streptavidin (SA, green) used to mark epithelial cells and help identify airways^79^; proliferative cells are labeled with Ki67 (red) to help identify tumors; MBP (yellow) is used to visualize subpopulation of nerve fibers. Scale bar = 50 µm. **d**, Body mass surveillance of animals up to 4 weeks following vagotomy or sham procedure (n = 5 animals per group). **e**, IVIS bioluminescence analysis of overall tumor growth in SCLC primary tumors measured weekly after vagotomy procedure (n = 11 sham and n = 9 vagotomy animals from 2 independent cohorts, n = 0.0041). Data represented as raw values of total flux. Arrowheads demonstrate animal endpoints. **f**, Quantification of percent of lung occupied by tumor in sham and denervated animals (n = 7 sham, n = 9 vagotomy, p = 0.0430). **g**, As in **f**, but for livers (n = 7 sham, n = 9 vagotomy, p = 0.0192). **h**, Gross image of livers harvested at the endpoint of the experiment from the sham operated (left) and denervated (right) animals. Scale bar = 1 cm. **i**, Representative immunofluorescence of nerve fibers (VAChT, yellow) around the early lung lesions (marked via Ki67, red) in RPR2-luc animals (left, 2–3 months since Cre administration, n = 4) compared to late-stage tumors (right, 8–9 months since Cre administration, n = 3). Streptavidin (SA, green) used to delineate airways. Scale bar = 100 µm. **j**, Quantification of data in **i** demonstrating decrease in innervation in late-stage tumors. **k**, Time course of tumor growth as measured by IVIS bioluminescence imaging in sham operated (grey) and denervated (vagotomy; red) animals that underwent surgery after initial tumor formation (n = 7 sham and n = 9 vagotomy animals from 2 independent cohorts). **l**, As in **k** for onset of liver metastasis as detected by IVIS imaging. **m**, Kaplan–Meier survival curve for sham operated (grey) and denervated (vagotomy; red) animals that underwent surgery after initial tumor formation (n = 7 sham and n = 9 vagotomy). Data are box plots for **a** (box defined by 25^th^ percentile, median, and 75^th^ percentile, whiskers extend to min/max, dots represent outliers), median +/- I.Q.R for **f,g,j**. Analysis with two-way ANOVA for **e**, two-sided unpaired Mann-Whitney test for **f, g, j**, Gehan-Breslow-Wilcoxon test for **k**, Log-rank (Mantel-Cox) test for **l, m**. All tests are two-tailed. *P < 0.05. Source data

We began by testing whether innervation supports SCLC growth in the lung. Recent studies have illustrated a role for neurotransmitter-mediated growth mechanisms in SCLC^33,37^, yet the role of vagal innervation in primary SCLC pathophysiology within the lung remains to be elucidated. Analysis of human primary SCLC samples^38^ revealed the expression of several neurotransmitter receptor genes, consistent with the idea that SCLC cells at the primary site possess the ability to respond to neuronal cues (Extended Data Fig. 1a). To further investigate the influence of neural input on SCLC pathobiology, we utilized the *Rb1*^*fl/fl*^;*Trp53*^*fl/fl*^;*p130*^*fl/fl*^ (RPR2; *p130* is also known as *Rbl2*), luciferase-expressing SCLC genetic mouse model^39^ (RPR2-luc). Tumours were induced in 8-week-old RPR2-luc mice by intratracheal administration of adenovirus expressing Cre under the control of the cytomegalovirus (CMV) promoter (Adeno-CMV-Cre). These mice form spontaneous SCLC tumours that recapitulate the genetics, histology, therapeutic response, time course of progression and metastatic nature of the human disease^40,41^. Examination of SCLC tumours taken from the mouse lung revealed innervation of malignant tissue by various nerve types, including parasympathetic (labelled by vesicular acetylcholine transporter protein), sympathetic (labelled by tyrosine hydroxylase protein) and sensory (a subpopulation of which is labelled by myelin basic protein) nerve fibres (Extended Data Fig. 1b). An abundance of nerve fibres was also evident in the vicinity of tumours metastatic to the liver in these mice (Extended Data Fig. 1c).

**Fig. 1:**
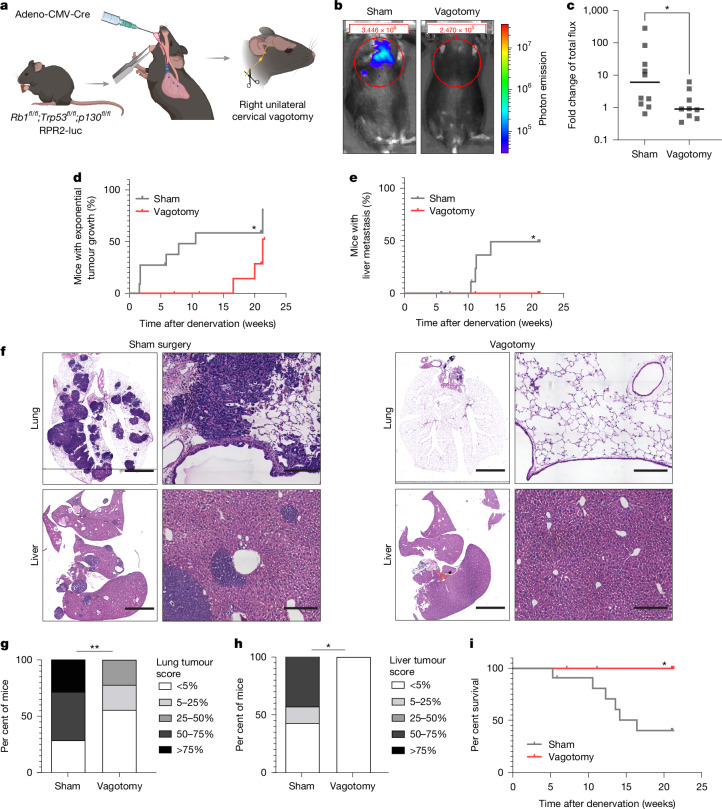
Vagal nerve innervation is critical for primary SCLC initiation and development. **a**, Experimental paradigm for unilateral cervical vagotomy in genetic mouse model of spontaneously forming SCLC (RPR2-luc). Created in BioRender. Savchuk, S. (2025) https://BioRender.com/5fwotqm. **b**, Representative in vivo imaging system (IVIS) image of RPR2-luc mice 10 weeks after sham or vagotomy procedure. Photon emission expressed as photons per second per cm^2^ per sr. The numbers on the images represent photon emission from the area selected within the red circles. **c**, Analysis of IVIS bioluminescence of overall tumour growth in SCLC primary tumours measured 10 weeks after vagotomy procedure (*n* = 11 sham and *n* = 9 vagotomy mice from 2 independent cohorts, *P* = 0.0465). Data are medians. **d**, Time course of tumour growth in RPR2-luc mice as measured by IVIS bioluminescence imaging after sham operation or vagotomy procedure. Events are recorded when mice begin to consistently show an increase in flux signal by tenfold between each weekly measurement (*n* = 11 sham and *n* = 9 vagotomy mice, *P* = 0.040). **e**, Time course of liver metastasis onset in RPR2-luc mice as detected by IVIS bioluminescence imaging after sham operation or vagotomy procedure (*n* = 11 sham and *n* = 9 vagotomy mice, *P* = 0.036). **f**, Representative haematoxylin and eosin (H&E) staining of lungs and livers isolated from sham-operated and denervated (vagotomy) RPR2-luc mice. Scale bars: 5,000 µm (left-hand images) and 250 µm (right-hand images). **g**, Quantification of lung tumour score (percentage of the organ occupied by the tumour) in sham-operated and denervated (vagotomy) RPR2-luc mice (*n* = 7 sham and *n* = 9 vagotomy mice, *P* = 0.005). **h**, As in **g**, for quantification of liver tumour score (*n* = 7 sham and *n* = 9 vagotomy mice, *P* = 0.019). **i**, Kaplan–Meier survival curve of RPR2-luc mice after either sham operation or denervation (vagotomy) (*n* = 11 sham and *n* = 9 vagotomy mice, *P* = 0.014). Mann–Whitney test (**c**); Gehan–Breslow–Wilcoxon test (**d**); log-rank (Mantel–Cox) test (**e**,**i**); Fisher’s exact test (**g**,**h**); all tests are two-tailed. **P < 0.01, *P < 0.05. Source data

To modulate innervation to the lung, we performed a unilateral cervical vagotomy in the RPR2 mouse model (Fig. 1a). Vagotomies or sham surgeries were performed approximately 2 months after the intratracheal administration of the Adeno-CMV-Cre vector but before the development of visible lesions^39^. Sham-manipulated and denervated mice tolerated the procedure well, displaying no postoperative weight loss or sickness behaviours (Extended Data Fig. 1d).

By 10 weeks post-denervation, a marked difference was evident in the overall tumour burden between the sham-manipulated (control) and denervated (vagotomy) groups (Fig. 1b,c). Longitudinal in vivo bioluminescent imaging revealed that the initiation of primary lung tumours and the appearance of liver metastasis were significantly delayed or not detected in the vagotomy cohort, a difference that continued throughout the remainder of the experiment (Fig. 1d,e and Extended Data Fig. 1e). At the endpoint (around 6 months post-vagotomy), these differences in overall tumour burden were histologically validated. Whereas sham-manipulated mice demonstrated an abundance of tumour sites throughout all lobes of the lung, the denervated mice illustrated minimal to no tumour burden (Fig. 1f,g and Extended Data Fig. 1f). Livers, the first site of metastasis, were found to be free of tumour in all denervated mice as assessed by both histological and gross analysis of the surface of the organ (Fig. 1f,h and Extended Data Fig. 1g,h). These results were independently evaluated by a board-certified pathologist. A caveat to note is that the lack of metastatic spread to the liver is influenced, at least in part, by the marked reduction in lung tumour burden in vagotomized mice. Finally, as the overall tumour burden was greatly reduced by vagotomy, we observed a substantial survival benefit for the mice that had been denervated, with all denervated mice surviving the full duration of the experiment compared with a median survival of 16 weeks for sham-manipulated mice (Fig. 1i).

**Extended Data Fig. 2:**
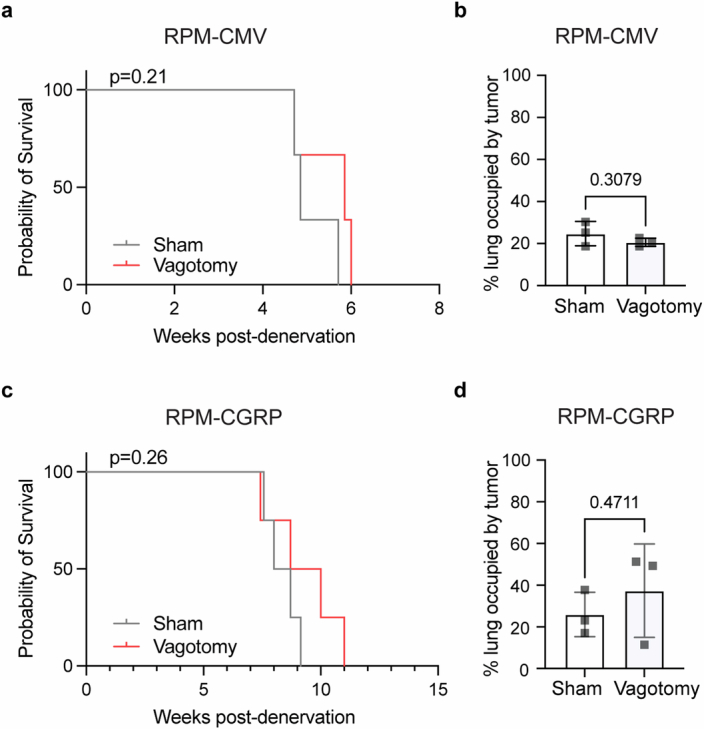
Denervation does not offer survival advantage for MYC-driven SCLC. **a**, Kaplan–Meier survival curve for sham operated (grey) and denervated (vagotomy; red) animals of RPM-CMV-Cre model (n = 3 sham and n = 3 vagotomy). **b**, Quantification of tumor burden by percent of lung occupied by tumor for animal cohort in **a. c**, As in **a** for RPM-CGRP-Cre model animals (n = 4 sham and n = 4 vagotomy). **d**, Quantification of tumor burden by percent of lung occupied by tumor for animal cohort in **c**. Data are mean ± s.e.m. for **b, d**. Analysis with Log-rank (Mantel-Cox) test for **a,c**, two-sided unpaired t-test for **b,d**. Source data

To then assess whether innervation was important to late-stage disease progression, we histologically evaluated innervation in early lesions compared with late-stage tumours. Compared with early in disease progression, more advanced tumours exhibited a significant reduction in nerve infiltration in regions of the tumour (Extended Data Fig. 1i,j). Concordantly, in contrast to early denervation, when vagotomy was performed 5–6 months after Cre administration in an independent cohort of mice, such reduction of tumour burden and survival benefit were not observed (Extended Data Fig. 1k–m). Similarly, tumour development was not inhibited by denervation in an aggressive form of SCLC driven by oncogenic MYC (*Trp53*^*fl/fl*^;*Rb1*^*fl/fl*^;*Myc*^*LSL/LSL*^ (RPM)^42^) where mice succumb to the tumour within 2 months (Extended Data Fig. 2). Together these findings comparing the role of innervation at early stages of tumour development to later stages and in more aggressive models suggest that vagal innervation of the primary tumour site (lung) has a critical role in SCLC initiation and development, and less of a role in tumour maintenance of advanced disease.

## Neurons in the intracranial SCLC microenvironment

**Fig. 2:**
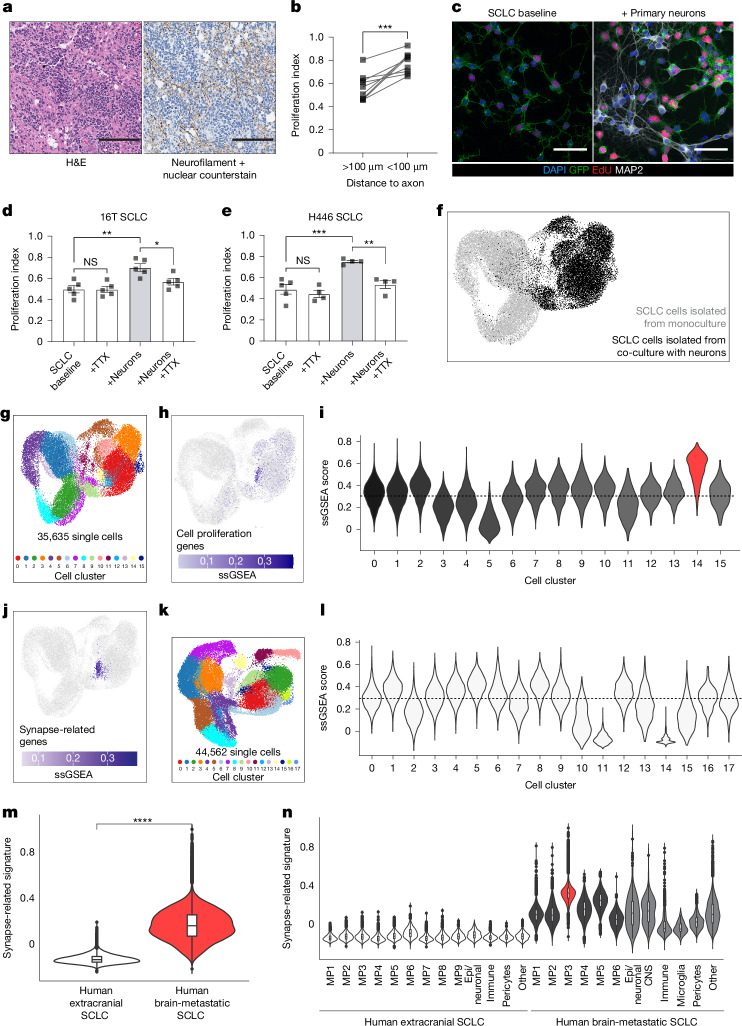
Neuronal activity promotes SCLC growth within the brain. **a**, Representative immunohistochemistry of human SCLC brain metastases. Left, H&E staining. Right, neurofilament (brown) with nuclear counterstain (blue). Scale bars, 150 µm. **b**, Proliferation index in regions of human SCLC brain metastases quantified less than or greater than 100 µm from axons (*n* = 9 patients, *P* = 0.0009). **c**, Representative images of mouse 16T SCLC cells (GFP) co-cultured with primary cortical neurons (MAP2). Proliferative cells are labelled with EdU. Scale bars, 50 µm. **d**, Quantification of data in **c** with or without addition of 1 µM TTX (*n* = 5 coverslips per condition, *P* = 0.0010). **e**, As in **d**, for human SCLC cells (H446, *n* = 4 coverslips per condition, *n* = 5 for baseline, *P* = 0.0003). **f**, Uniform manifold approximation and projection (UMAP) embedding of scRNA-seq profiles of mouse 16T SCLC cells isolated from monoculture or co-culture with primary mouse neurons. **g**, Distribution of SCLC cells in **f** on the UMAP embedding plot. **h**, Gene set enrichment analysis (GSEA) of the 16 populations identified in **g** reveals a distinct cluster among the SCLC cells isolated from neuron co-cultures that is enriched for proliferation-related genes (all gene signatures in Supplementary Table 1). ssGSEA, single-sample GSEA. **i**, Quantification of ssGSEA scores for synapse-related gene signature across the 16 clusters in **g** detects significant upregulation in cluster 14 (red, statistical testing in Supplementary Table 2). **j**, Visualization of cell clusters enriched for synapse-related genes among SCLC cells isolated from neuronal co-culture (cluster 14). **k**, Distribution of SCLC cells treated with 1 µM TTX in monoculture or neuron co-culture (Extended Data Fig. 4f) on the UMAP embedding plot. **l**, As in **i** but in the presence of 1 µM TTX (the 18 clusters in **k**). **m**, Expression of synapse-related genes in cells isolated from patient lung primary or recurrent or non-brain-metastatic lesions^45^ (*n* = 16) versus cells from patient SCLC brain metastases (*n* = 12, *P* < 0.0001). **n**, Expression of synapse-related genes across the cell types and malignant cell metaprogrammes (MP1–MP9) detected in patient lung primary, recurrent, non-brain-metastatic lesions (*n* = 16) or SCLC brain metastases (*n* = 12 patients). CNS, central nervous system; epi, epithelial. Data are mean ± s.e.m. (**d**,**e**); violin plots (**i**,**l**); violin and box plots (**m**,**n**). In box plots, the centre line is the median, box edges delineate 25th and 75th percentiles and whiskers extend to minimum and maximum values; dots represent outliers. Paired *t*-test (**b**); two-way ANOVA (**d**,**e**); one-way ANOVA with Tukey correction (**i**,**l**); pairwise Wilcoxon rank sum test (**m**). All tests are two-tailed. ****P < 0.0001, ***P < 0.001; NS, not significant. Source data

**Extended Data Fig. 3:**
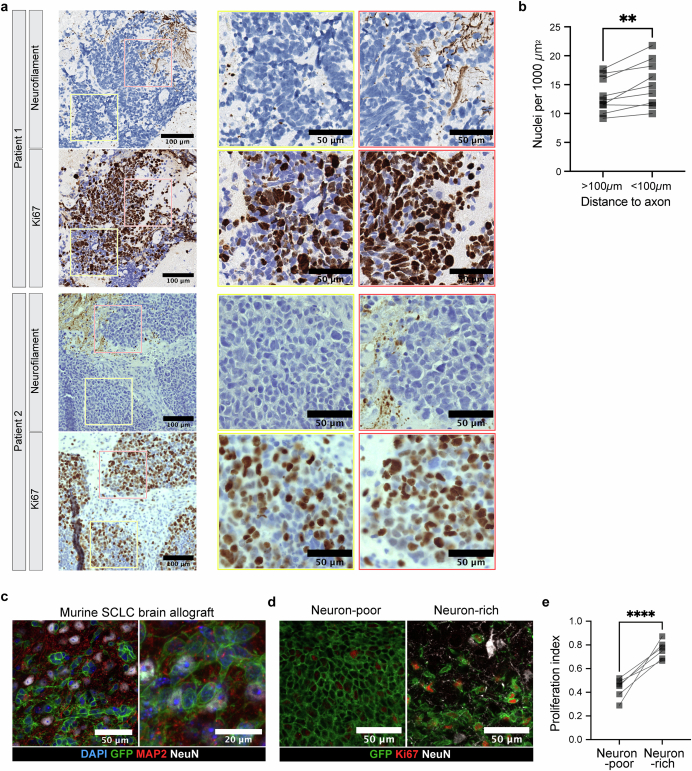
Microenvironmental dynamics within intracranial SCLC tumors. **a**, Representative neurofilament (top) and Ki67 (bottom) immunohistochemistry of two human samples of SCLC brain metastasis in regions within (red box, zoomed in on right panel) and outside (yellow box, zoomed in on middle panel) 100 µm distance from neurofilament. Scale bar on left = 100 µm; scale bar on middle and right = 50 µm. **b**, Comparison of local nuclear density as a measure of proliferative history in human SCLC brain metastases for n = 9 patients. Regions are grouped by distance to axons immunostained for neurofilament (less than or greater than 100 µm, p = 0.0044). **c**, Representative immunofluorescent images of murine SCLC brain allografts (GFP, green) with neurons labeled with MAP2 (red) and NeuN (white). Scale bar = 50 µm (left), 20 µm (right). **d**, Representative images of proliferation rate (Ki67, red) of murine SCLC allografts (GFP, green) assessed in regions deprived of (left) or enriched for (right) neurons. Scale bar = 50 µm. **e**, Quantification of data in **d**, illustrating increased proliferation in neuron-rich compared to neuron-poor areas (n = 6 animals, p < 0.0001). Analysis with two-tailed paired t-test for **b, e**. ****P < 0.0001, ***P < 0.001, **P < 0.01, *P < 0.05. Source data

Given the substantial role of innervation in primary SCLC as demonstrated above and the high propensity of SCLC to form intracranial metastases, we next evaluated the interactions between neurons and SCLC cells in the brain. We first analysed metastatic SCLC brain tissue samples from nine human patients. Immunohistochemical neurofilament staining revealed that regions of the tumour mass demonstrated extensive axons intermingled with malignant cells (Fig. 2a and Extended Data Fig. 3a), whereas other regions exhibited little to no axonal infiltration. Quantifying the proliferation index (fraction of Ki67^+^ cells) of SCLC, we found that malignant cells that were closer to axons (within 100 µm) exhibited increased rates of proliferation and higher nuclear density (Fig. 2b and Extended Data Fig. 3b), suggesting a possible functional role of neuron–SCLC interactions within the brain.

As there is no reliable model of spontaneous brain metastasis in any of the existing intracardiac or intravenous SCLC cell line administration models or genetic mouse models of SCLC^36,43^, we used mouse intracranial allografts, developed from isolation of metastases from the genetic models^44^ or patient samples, to test potential functional influences of neuronal activity on the growth of intracranial SCLC. This enabled placement of the malignant cells into specific regions of interest, allowing direct manipulation of distinct neural circuits involving the tumour. It is important to note that this is not a model of metastatic initiation, but rather one of established intracranial tumour growth. These brain allografts exhibited neurons present within the tumour mass, similar to the human samples described above, especially in areas of the tumour periphery (Extended Data Fig. 3c). Consistent with the human patient samples, we found the proliferation index of cells in neuron-rich peripheral tumour regions to be significantly higher than in neuron-poor areas within the periphery of the mass (Extended Data Fig. 3d,e).

## Neuron–SCLC interactions in the brain

**Extended Data Fig. 4:**
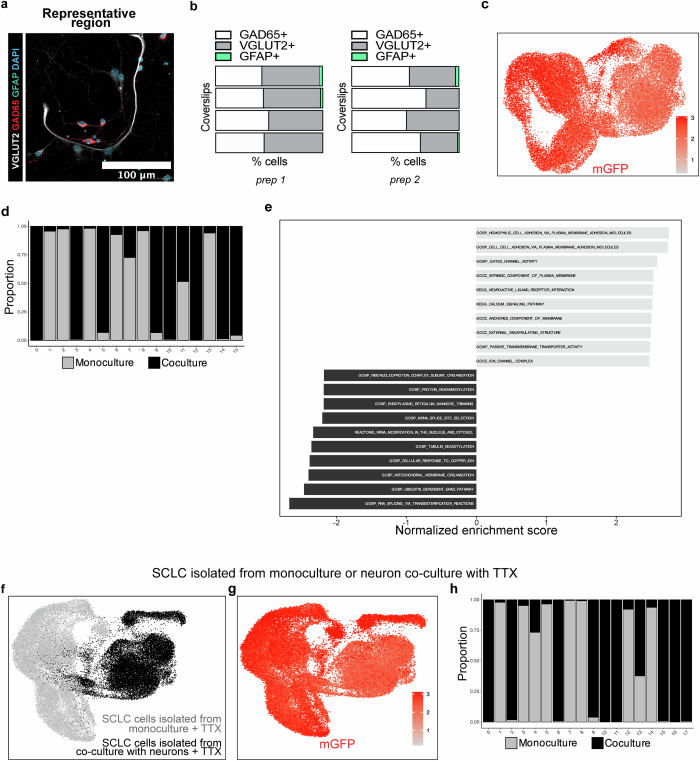
Single-cell RNA sequencing of SCLC cells after co-culture with neurons, either with or without addition of tetrodotoxin, reflects distinct subpopulations of cells that cluster separately from SCLC cells in monoculture. **a**, Representative immunofluorescence of neuronal populations present in primary neuronal co-cultures demonstrating presence of glutamatergic (VGLUT2, white) and GABA-ergic (GAD65, red) neurons and absence of astrocytes (GFAP, green). Scale bar = 100 µm. Data from 2 independent experiments. **b**, Quantification of data in **a**, demonstrating approximately equal proportions of glutamatergic and GABA-ergic neurons. **c**, Expression of membrane GFP (mGFP) in 16T SCLC cells grown in monoculture or isolated from co-culture with primary murine neurons. **d**, Composition of the 16 clusters depicted in Fig. 2g as percentage of cells isolated from the monoculture (grey) or co-culture (black) conditions. **e**, Gene Ontology (GO) enrichment analysis for the top pathways expressed by cells in cluster 14. **f**, UMAP embedding of single cell RNA-seq profiles of murine 16T SCLC cells isolated from monoculture (grey) or co-culture (black) with primary murine neurons in the presence of 1 µM TTX. 44,562 cells were analyzed from 3 biological replicates. **g**, Expression of membrane GFP (mGFP) in cells from **f**. **h**, Composition of the 18 clusters depicted in Fig. 2k as percentage of cells isolated from the monoculture (grey) or co-culture (black) in the presence of TTX conditions.

To probe the interactions between SCLC and neuronal activity, we utilized co-culture systems in which cortically derived mouse neurons, representing a mixture of glutamatergic and GABAergic subpopulations (Extended Data Fig. 4a,b), were cultured together with either mouse 16T SCLC cells or human H446 SCLC cells. In both cases, the presence of active neurons significantly increased the proliferation rate of SCLC cells in co-culture (Fig. 2c–e). This effect was abrogated with the addition of tetrodotoxin (TTX), a voltage-gated sodium channel blocker that inhibits neuronal action potentials (Fig. 2d,e).

We next examined the transcriptional phenotypic changes in SCLC cells following exposure to neurons. We performed single-cell RNA sequencing (scRNA-seq) of the SCLC cells (16T-GFP) isolated from neuronal co-culture using fluorescence-activated cell sorting (FACS) (Extended Data Fig. 4c). When compared to cells from SCLC monoculture, the co-cultured cells clustered separately with distinct subpopulations primarily composed of either mono-cultured or co-cultured SCLC cells (Fig. 2f,g and Extended Data Fig. 4d). In line with the proliferative effect seen in areas of neuronal infiltration, we found that upregulation of a cell proliferation signature was a prominent effect of neuronal co-culture (Fig. 2h).

## Activity-mediated synaptic signature in SCLC

In addition to the upregulation of the proliferative signature, Gene Ontology (GO) analysis identified a distinct cluster of cells isolated from neuronal co-culture that are defined by GO terms linked to synapse formation and neurotransmitter receptors and enriched for a synapse-related gene signature (cluster 14; Fig. 2i,j, Extended Data Fig. 4e and Supplementary Tables 1 and 2). To determine whether this synapse-related gene expression enrichment after exposure to neurons was mediated by neuronal activity, we performed a separate experiment comparing mono-cultured and co-cultured cells in the presence of TTX (Fig. 2k and Extended Data Fig. 4f–h). In the absence of neuronal activity, no malignant subpopulations were found to be enriched for the synapse-associated gene signature, indicating an activity-dependent mechanism of malignant cellular plasticity (Fig. 2l).

**Extended Data Fig. 5:**
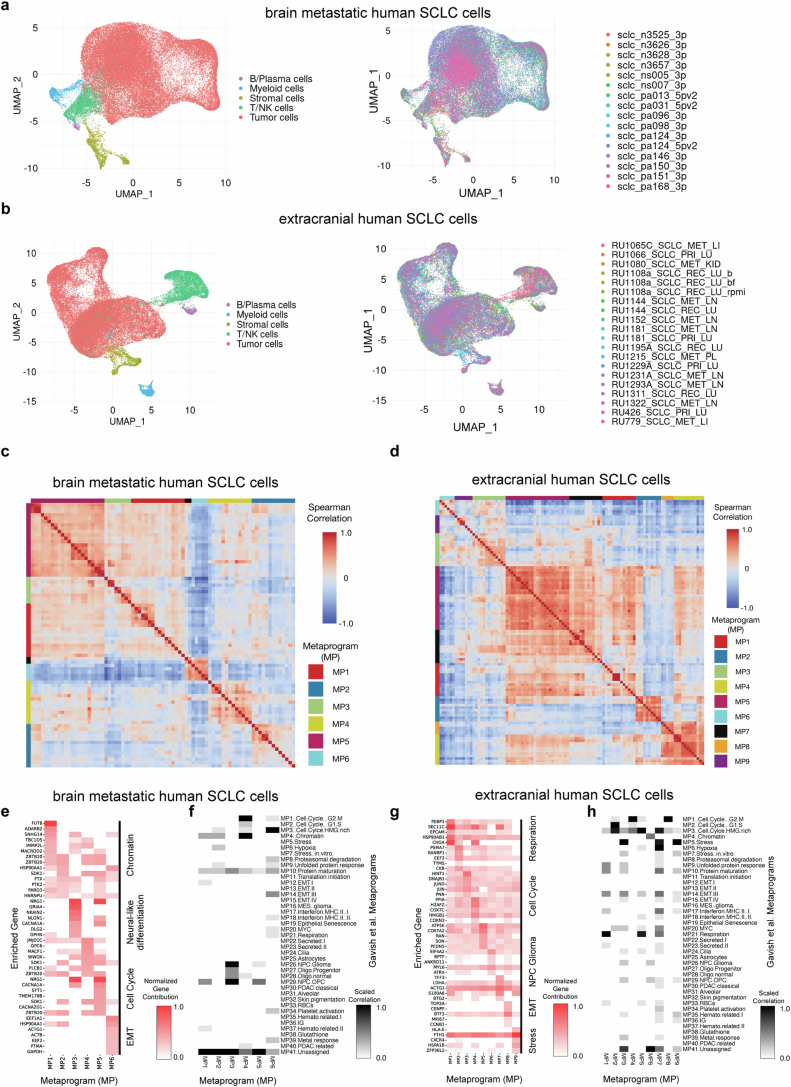
Human brain metastatic SCLC contains a subpopulation enriched for neural gene signatures. **a**, Integrated UMAP embeddings of all cells from a cohort of n = 12 patients with SCLC brain metastases, colored by their assigned cell-types (left) or patient (right). **b**, as in **a**, but of cells from n = 16 patients with lung primary, recurrent, and non-brain-metastatic SCLC lesions (data made publicly available by Chan et al., 2021). **c**, Spearman correlation of cell programs classifying malignant cells in **a**, grouped into 6 distinct metaprograms (indicated by boxes within the co-correlation plot). **d**, as in **c**, but for malignant cells in **b** grouped into 9 distinct metaprograms. **e**, Normalized gene contribution of top defining genes (rows) for each respective metaprogram in the brain-metastatic cohort (columns). Annotated biological functions corresponding to these genes, individual metaprograms, or groups of metaprograms are shown on the right. Metaprogram 3 is defined by genes driving neural-like differentiation. **f**, Scaled correlation of brain-metastatic cohort metaprograms (columns) with annotated transcriptional hallmarks of tumor heterogeneity derived by Gavish et al.^46^ (rows). Metaprogram 3 demonstrates a strong correlation with neural-like hallmarks. **g**, as in **e** for the primary, recurrent, and non-brain metastatic cohort. **h**, as in **f** for the primary, recurrent, and non-brain metastatic cohort.

To determine whether this malignant signature could be detected in human biopsies, we collected SCLC brain metastases from 12 patients and analysed them using single-nucleus RNA sequencing (Extended Data Fig. 5a). As patients with SCLC do not routinely undergo tumour resection, there are no matched primary and brain-metastatic tissue datasets. We therefore compared the single-nucleus data from our SCLC brain metastasis tissue to a publicly available single-cell dataset^45^ incorporating cells from primary SCLC tumours and various unmatched metastatic disease sites including lung, pleura, lymph nodes, liver and kidney (Extended Data Fig. 5b). We found that compared with all other disease sites, SCLC cells in the brain were enriched for the synaptic signature (Fig. 2m). Further, unbiased clustering of human brain-metastatic cells revealed that SCLC cells within the brain could be grouped into six distinct metaprogrammes (Extended Data Fig. 5c). In line with the results from our co-culture dataset (Fig. 2i,j), we found that one specific metaprogramme (MP3) was defined by genes comprising neural-like transcriptional programmes (Fig. 2n and Extended Data Fig. 5e). When further assessed against previously defined pan-cancer metaprogrammes^46^, MP3 correlated with programmes associated with neural and glial precursor-like signatures (Extended Data Fig. 5f). Conversely, we did not find any enrichment of these programmes in SCLC cells from extracranial disease sites (Extended Data Fig. 5d,g,h), suggesting that compared with elsewhere in the body, intracranial SCLC is specifically enriched for neural gene expression programmes.

## Synaptic integration of SCLC in the brain

**Fig. 3:**
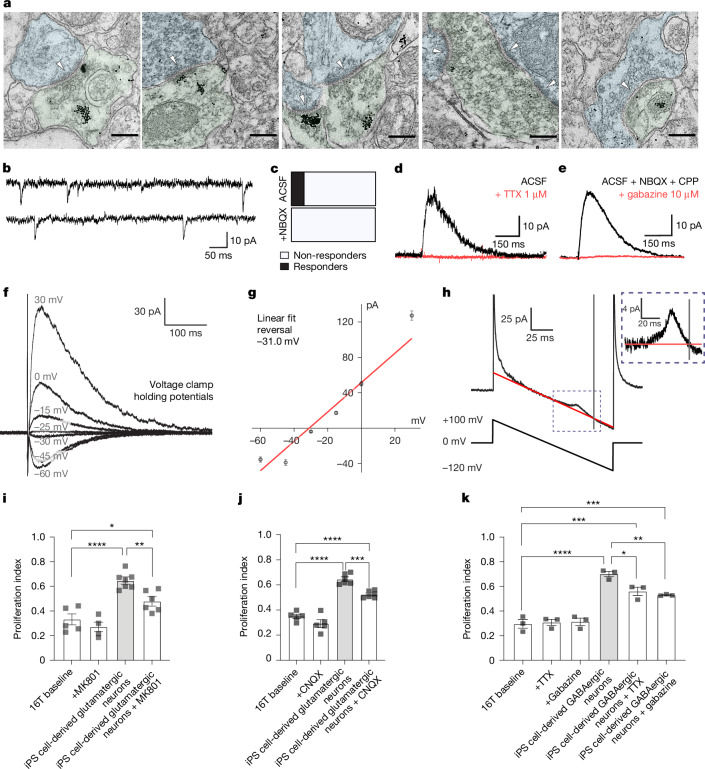
SCLC cells exhibit synaptic currents that drive tumour progression. **a**, Immuno-electron microscopy of 16T-GFP SCLC cells allografted to mouse hippocampus. Black dots represent immunogold particles labelling GFP (tumour cells). Post-synaptic density in GFP^+^ tumour cells (pseudo-coloured green), synaptic cleft and clustered synaptic vesicles in apposing pre-synaptic neuron (blue) identify synapses (white arrowheads). Scale bars, 200 nm. **b**, Representative recordings of sEPSCs in allografted 16T SCLC cells. **c**, Fraction of SCLC cells demonstrating spontaneous currents illustrated in **b** at baseline (artificial cerebrospinal fluid (ACSF), 8 out of 53 cells) or with the addition of 10 µM NBQX (bottom, 0 out of 27 cells). **d**, Representative traces of neuronal activity-dependent synaptic currents evoked in SCLC (*n* = 27 out of 49 cells), blocked after application of 1 µM TTX. **e**, Representative traces of neuronal activity-dependent evoked SCLC currents before and after application of 10 µM gabazine (*n* = 3 out of 3 cells). **f**, Representative trace of 16T SCLC cell currents in response to local GABAergic stimulation in the presence of glutamatergic inhibitors in perforated-patch recordings using gramicidin D at varying membrane potentials. **g**, Current–voltage relationship of GABAergic stimulation-induced current in 16T SCLC cells recorded with perforated-patch electrophysiology. Reversal potential of GABA was −27.3 ± 5.5 mV (−31.0 mV based off linear fit in example trace); *n* = 6 cells across 3 mice. **h**, Cell-attached measurement of resting membrane potential of SCLC cells. Current traces recorded during voltage ramp are shown in black. The red line is the extrapolated leak current from a linear fit and vertical grey line indicates the intersection of the voltage-activated K^+^ current with the leak current, yielding the resting membrane potential of −72 ± 7 mV (*n* = 7 cells). **i**, Proliferative index of mouse 16T SCLC-A subtype cells co-cultured with human iPS cell-derived glutamatergic neurons reveals increased proliferation in co-culture, abrogated by the addition of 50 µM MK801 (NMDA receptor inhibitor for glutamate, *n* = 5 coverslips for baseline condition, *n* = 4 for MK801, *n* = 7 for co-culture and *n* = 6 for co-culture with MK801, *P* < 0.0001). **j**, As in **i**, but with 50 µM CNQX (AMPA receptor inhibitor for glutamate) (*n* = 5 coverslips per condition, *P* < 0.0001). **k**, Proliferative index of mouse SCLC-A subtype 16T cells co-cultured with human iPS cell-derived GABAergic neurons with or without addition of 1 µM TTX or 20 µM gabazine (GABA_A_ receptor inhibitor) reveals that increased proliferation in co-culture is abrogated by TTX or gabazine (*n* = 3 coverslips per condition, *P* < 0.0001). Data are mean ± s.e.m. (**i**–**k**). Two-way ANOVA (**i**–**k**). All tests are two-tailed. Source data

**Extended Data Fig. 6:**
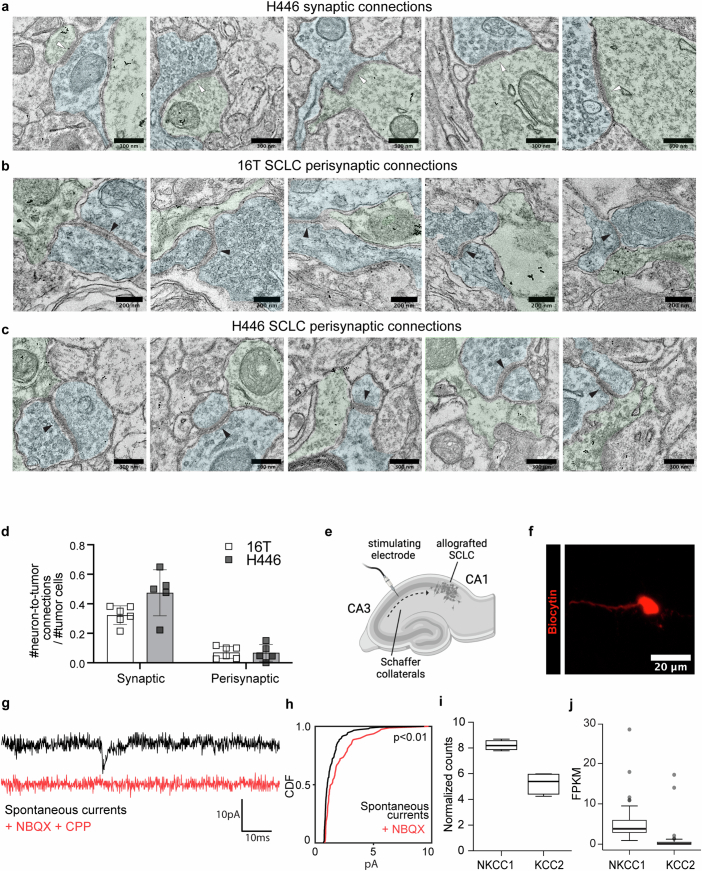
Electrical currents in SCLC pathogenesis. **a**, Immuno-electron microscopy of H446-GFP SCLC allografted to mouse hippocampus. Black dots represent immunogold particles labeling GFP (tumor cells). Post-synaptic density in GFP+ tumor cells (pseudo-colored green), synaptic cleft, and clustered synaptic vesicles in apposing presynaptic neuron (pseudo-colored blue) identify synapses (white arrowheads). Scale bar = 300 nm. **b**, Immuno-electron microscopy of 16T-GFP SCLC allografted to mouse hippocampus to demonstrate perisynaptic SCLC connections. Post-synaptic density in GFP-negative neuron (pseudocolored blue), synaptic cleft, and clustered synaptic vesicles in apposing presynaptic neuron identify neuron-to-neuron synapses (black arrowheads). Tumor cells in perisynaptic position are pseudocolored green. Scale bar = 200 μm. **c**, as in **b**, but with H446-GFP SCLC cells. Scale bar = 300 μm. **d**, Quantification of synaptic and perisynaptic neuron-to-tumor connections in murine 16T and human H446 SCLC cells. **e**, Experimental paradigm for acute slice electrophysiology. GFP + SCLC cells (grey) allografted into the mouse hippocampal CA1 region with CA3 Schaffer collateral afferent stimulation. Created in BioRender. Savchuk, S. (2025) https://BioRender.com/5fwotqm. **f**, Biocytin-filled (red) SCLC cell allografted to CA1 region of mouse hippocampus. Scale bar = 20 μm. **g**, Representative recordings of spontaneous currents in allografted SCLC cells at baseline (black) or after addition of 10 µM glutamatergic inhibitors NBQX and CPP (red). **h**, Cumulative probability density function (CDF) of membrane current of SCLC cells recorded at baseline (n = 8) and after application of glutamate receptor antagonists (n = 15). **i**, Expression of K+/Cl− co-transporter genes in SCLCs cell lines (n = 4 samples). **j**, Expression of K+/Cl− co-transporter genes in human samples of primary SCLC (n = 81 patients). Data are plotted as mean ± s.e.m. for **d**, box-and-whisker plots for **i, j**, (box defined by 25^th^ percentile, median, and 75^th^ percentile, whiskers extend to min/max, dots represent outliers). Analysis with 2-sided t-test, Wilcoxon rank sum, and Kolmogorov-Smirnov for **h**. Source data

We next assessed whether neurotransmitter-mediated signalling occurred through the formation of direct synapses between SCLC cells and neurons in the tumour microenvironment. Examination of ultrastructural interactions in allografted or xenografted brain tissue using electron microscopy revealed clear synaptic structures (Fig. 3a and Extended Data Fig. 6a). Immunogold labelling of GFP-tagged 16T or H446 SCLC cells unambiguously identified the malignant cells at post-synaptic sites, confirming that these cancer cells structurally participate in neuron–SCLC synapses (approximately 3–5 synapses per 10 SCLC cells; Extended Data Fig. 6d). Although less frequent, immuno-electron microscopy also identified malignant cells in a perisynaptic position juxtaposed to normal neuron–neuron synapses (Extended Data Fig. 6b–d) consistent with the ‘pseudo-tripartite’ synapses previously described in breast cancer brain metastases^17^.

Having established the presence of structural neuron-to-SCLC synaptic interactions, we next evaluated synaptic responses of SCLC to neuronal activity electrophysiologically. GFP-labelled SCLC 16T cells were allografted into the CA1 region of the hippocampal circuit, an experimentally tractable circuit that is amenable to electrophysiological interrogation of neuron-to-cancer synapses^2,16^ (Extended Data Fig. 6e,f). After a period of engraftment, we prepared acute hippocampal slices for whole-cell electrophysiological recordings of GFP^+^ SCLC cells. Electrophysiological recordings in voltage clamp at −70 mV of individual SCLC cells revealed the presence of spontaneous excitatory post-synaptic currents (sEPSCs) in a subpopulation of cancer cells (approximately 22% of SCLC cells; Fig. 3b). In the presence of NBQX (2,3-dihydroxy-6-nitro-7-sulfamoyl benzo[f]quinoxaline), a glutamatergic (AMPA (α-amino-3-hydroxy-5-methyl-4-isoxazole propionic acid)) receptor blocker, these sEPSCs were no longer detected and variance in membrane current was significantly decreased (Fig. 3c and Extended Data Fig. 6g,h), indicating that these are spontaneous currents arising from glutamatergic input and consistent with functional synapses between glutamatergic neurons and SCLC cells mediated by an AMPA subtype of glutamate receptors.

To assess for action potential evoked responses, we stimulated CA3 Schaffer collateral and commissural axons, inputs to CA1, while simultaneously recording from SCLC cells in CA1 using a low-chloride internal solution. At voltage clamp of −70 mV to minimize GABA currents, evoked responses were very rarely seen (less than 5%). When held at 0 mV to minimize glutamatergic currents, a high proportion of SCLC cells (approximately 55%) exhibited currents evoked by neuronal stimulation. These currents were blocked with the addition of TTX to prevent action potentials (Fig. 3d). To determine whether these currents occurred in response to GABAergic input, recordings were performed in the presence of NBQX and CPP (3-(2-carboxypiperazin-4-yl)propyl-1-phosphonic acid) to inhibit AMPAR and *N*-methyl-d-aspartate (NMDA) receptor-mediated currents, respectively. In the absence of glutamatergic activity, electrical stimulation consistently resulted in currents that were blocked by addition of the GABA_A_ receptor inhibitor gabazine (Fig. 3e), indicating GABAergic synaptic currents in SCLC cells. Given that GABA can act as either a depolarizing or hyperpolarizing neurotransmitter depending on intracellular chloride concentration, we assessed its role in the context of malignant SCLC cells without altering intracellular chloride concentration. To determine the physiological reversal potential of GABA currents in SCLC cells, we recorded responses to stimulation of GABAergic synapses (via local stimulation in the presence of glutamatergic inhibitors) with perforated-patch recordings from SCLC cells using gramicidin D. GABAergic synaptic currents in SCLC cells reversed at −27.3 ± 5.5 mV (Fig. 3f,g), which corresponds to an intracellular chloride concentration of 46.8 mM. This is consistent with expression of K^+^/Cl^−^ co-transporters in SCLCs, with the *NKCC1* gene overexpressed in relation to *KCC2* (Extended Data Fig. 6i,j), driving the high intracellular concentration of chloride^47^. Separate cell-attached measurements revealed SCLC baseline resting membrane potential to be approximately −72 mV (Fig. 3h), more negative than the −27.3 mV chloride equilibrium potential; thus, opening of GABA-gated chloride channels causes SCLC depolarization rather than hyperpolarization. Together, these electrophysiological studies reveal activity-induced membrane depolarization and neurotransmitter-mediated signalling from neurons to SCLC cells in the brain.

## Neurotransmitter-mediated signalling in SCLC growth

**Extended Data Fig. 7:**
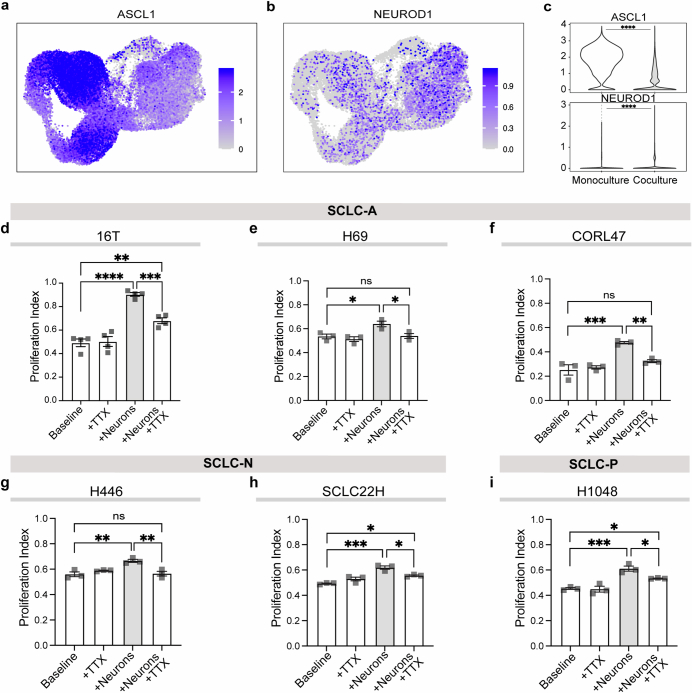
Human iPSC-derived glutamatergic neurons drive activity-dependent proliferation of multiple SCLC subtypes. **a**, Expression of *ASCL1* in 16T SCLC cells grown in monoculture or isolated from co-culture with primary murine neurons and processed for single cell RNA sequencing (see Fig. 2f). **b**, As in **a** for *NEUROD1*. **c**, Quantification of *ASCL1* and *NEUROD1* in 16T SCLC cells grown in monoculture or neuron co-culture (data from **a**, **b**, p < 0.0001). **d**, Quantification of proliferative index of murine 16T SCLC-A subtype cells co-cultured with human iPSC-derived glutamatergic neurons reveals increased proliferation in co-culture, abrogated by the addition of 1 µM TTX (n = 4 coverslips per condition, p < 0.0001). **e**, As in **d**, but human H69 SCLC-A subtype cells were used (n = 3 coverslips per condition, p = 0.0110). **f**, As in **d**, but human CORL47 SCLC-A subtype cells were used (n = 3 coverslips per condition, p = 0.0006). **g**, As in **d**, but human H446 SCLC-N subtype cells were used (n = 3 coverslips per condition, p = 0.0030). **h**, As in **d**, but human SCLC22H SCLC-N subtype cells were used (n = 3 coverslips per condition, p = 0.0002). **i**, As in **d**, but human H1048 SCLC-P subtype cells were used (n = 3 coverslips per condition, p = 0.0003). Data are violin plot for c, mean ± s.e.m for **d-i**. Analysis with Wilcoxon rank sum test for c, 2-way ANOVA for **d-i**. All tests are two-tailed. ****P < 0.0001, ***P < 0.001, **P < 0.01, *P < 0.05.

**Extended Data Fig. 8:**
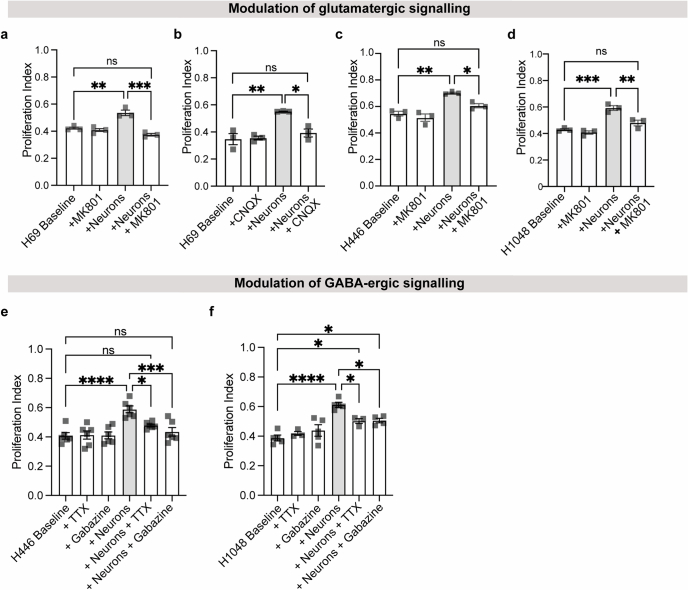
Glutamatergic and GABA-ergic signaling drives proliferation of multiple SCLC subtypes via neurotransmitter-mediated signaling pathways. **a**, Quantification of proliferative index of human SCLC-A subtype H69 cells co-cultured with human iPSC-derived glutamatergic neurons with or without addition of 50 µM MK801 (inhibitor of NMDA receptor for glutamate) reveals increased proliferation in co-culture abrogated by MK801 (n = 3 coverslips per condition, p = 0.0002). **b**, As in **a**, but 50 µM CNQX (inhibitor of AMPA receptor for glutamate) was used (n = 3 coverslips per condition, p = 0.0017). **c**, As in **a**, but human SCLC-N subtype H446 cells were used (n = 3 coverslips per condition, p = 0.0005). **d**, As in **a**, but human SCLC-P subtype H1048 cells were used (n = 3 coverslips per condition, p = 0.0001). **e**, Quantification of proliferative index of human SCLC-N subtype H446 cells co-cultured with human iPSC-derived GABA-ergic neurons with or without addition of 1 µM TTX or 20 µM Gabazine (GABA-A receptor inhibitor) reveals increased proliferation in co-culture abrogated by TTX and Gabazine (n = 4–6 coverslips per condition, p < 0.0001). **f**, As in **e**, but human SCLC-P subtype H1048 cells were used (n = 3–5 coverslips per condition, p < 0.0001). Data are mean ± s.e.m. Analysis with 2-way ANOVA. ****P < 0.0001, ***P < 0.001, **P < 0.01, *P < 0.05.

Given the distinct synaptic input from glutamatergic and GABAergic neuronal populations, we then examined whether direct neurotransmitter-mediated signalling contributes to activity-mediated increases in SCLC proliferation. We utilized induced pluripotent stem cell (iPS cell)-derived neurons to establish isolated glutamatergic or GABAergic neuronal populations. These neurons were cultured with a panel of human cell lines that represent clinically and molecularly distinct subtypes of SCLC, conventionally defined by high expression of specific transcriptional regulators ASCL1 (SCLC-A), NEUROD1 (SCLC-N) or POU2F3 (SCLC-P)^41^. Notably, our scRNA-seq analysis revealed downregulation of ASCL1 expression and upregulation of NEUROD1 expression in response to neuronal activity (Extended Data Fig. 7a–c). Co-cultures of these distinct SCLC subtypes and neuronal populations revealed that glutamatergic and GABAergic neurons elicited a proliferative effect across SCLC, independent of transcriptional phenotype (Fig. 3i–k and Extended Data Figs. 7 and 8). We added either TTX (a broad neuronal activity blocker) or the specific neurotransmitter receptor inhibitors MK801 (NMDA receptor inhibitor), CNQX (AMPA/kainate receptor inhibitor) or gabazine (GABA receptor inhibitor) to neuron–SCLC co-cultures and quantified SCLC proliferation. The addition of each of these inhibitors reduced the neuronal activity-induced proliferation of the SCLC cells (Extended Data Figs. 7 and 8), indicating involvement of both glutamatergic and GABAergic signalling in the SCLC proliferative response to neuronal activity.

**Extended Data Fig. 9:**
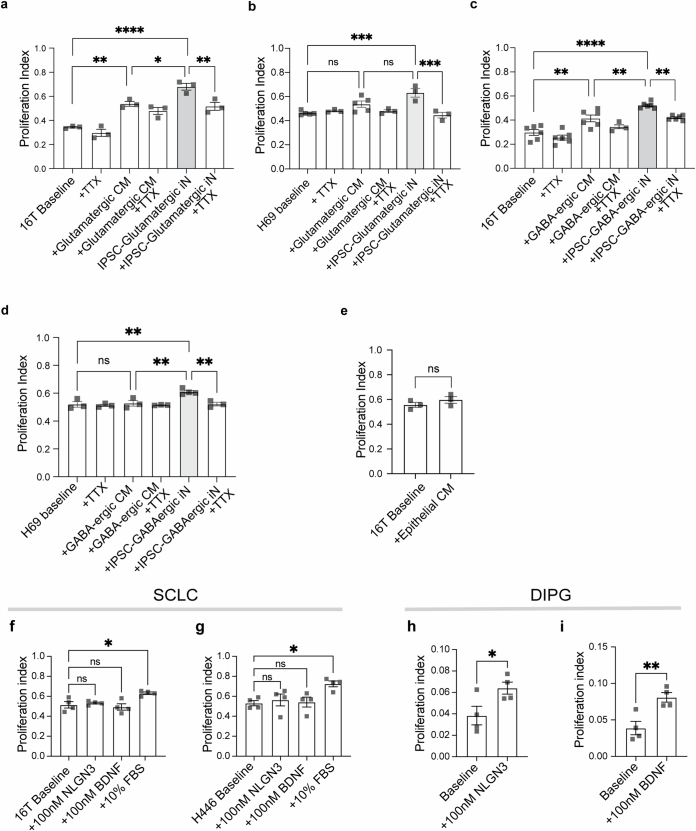
Neuron-conditioned media increases proliferation of SCLC, but not to the extent of direct co-culture. **a**, Quantification of proliferative index of murine 16T SCLC cells exposed to conditioned media (CM) from or directly co-cultured with iPSC-derived glutamatergic neurons with or without addition of 1 µM TTX (n = 3 coverslips per condition, p < 0.0001). Conditioned media alone renders a partial increase in SCLC proliferation compared to baseline, but not to the full effect of direct co-culture (grey). **b**, As in **a**, but human H69 SCLC cells were used (n = 3–5 coverslips per condition, p = 0.0003). **c**, As in **a**, but iPSC-derived GABA-ergic neurons were used (n = 3–6 coverslips per condition, p < 0.0001). **d**, As in **c**, but human H69 SCLC cells were used (n = 3–4 coverslips per condition, p = 0.0007). **e**, Quantification of proliferative index of murine 16T SCLC cells exposed to conditioned media (CM) from lung epithelial cells (n = 3 coverslips per condition, p = 0.9010. **f**, Quantification of proliferative index of murine 16T SCLC cells at baseline or when treated with 100 nM neuroligin 3 (NLGN3) or 100 nM brain derived neurotrophic factor (BDNF). 10% FBS condition is used as positive control (n = 4 coverslips per condition, p = 0.9403 for NLGN3, p = 0.8969 for BDNF). **g**, As in **f** but for human H446 SCLC cells (n = 4 coverslips per condition, p = 0.9387 for NLGN3, p = 0.9969 for BDNF). **h**, Quantification of proliferative index of patient derived glioma cells (SU-DIPGVI) at baseline or treated with 100 nM NLGN3 (n = 4 coverslips per condition, p = 0.0483). **i**, as in **h** for glioma cells treated with 100 nM BDNF (n = 4 coverslips per condition, p = 0.0096). Data are mean ± s.e.m. Analysis with one-way ANOVA for **a, b, c, d, f, g**, two-tailed unpaired t-test **e, h, i**. ****P < 0.0001, ***P < 0.001, **P < 0.01, *P < 0.05.

To determine whether these effects of neurons on SCLC were due to direct contact-mediated or paracrine signalling mechanisms, we applied conditioned medium from either glutamatergic or GABAergic neurons in the presence or absence of TTX to mouse or human SCLC cells in culture. Conditioned medium from neurons elicited either no increase or a partial increase in SCLC proliferation that was significantly less than the larger effect elicited by direct neuronal co-culture (Extended Data Fig. 9a–d). Conditioned medium taken from lung-derived epithelial cells had no proliferative effect (Extended Data Fig. 9e). These results suggest that paracrine factors secreted by neurons may contribute, but alone are insufficient to wholly explain the full proliferative effect of direct co-culture. Thus, contact-mediated interactions—such as synaptic communication—account for an important component of the growth-promoting effects of neurons on SCLC cells. We then assessed whether the known activity-dependent paracrine factors NLGN3 and BDNF, which we have shown promote glioma growth^1,13,14,16^, similarly induce proliferation in SCLC. Neither of these paracrine factors were found to affect the proliferation of the lung cancer cells (either 16T or H446) in vitro, in contrast to a clear growth-promoting effect in patient-derived glioma cultures (Extended Data Fig. 9f–i). These results are in line with previous studies indicating that NLGN3 is not critical for the growth of breast cancer brain metastases in vivo^15^, and highlight the need to disentangle the distinct activity-dependent mechanisms that influence growth across cancers arising from various tissue origins.

## Circuit activity drives growth of intracranial SCLC

**Fig. 4:**
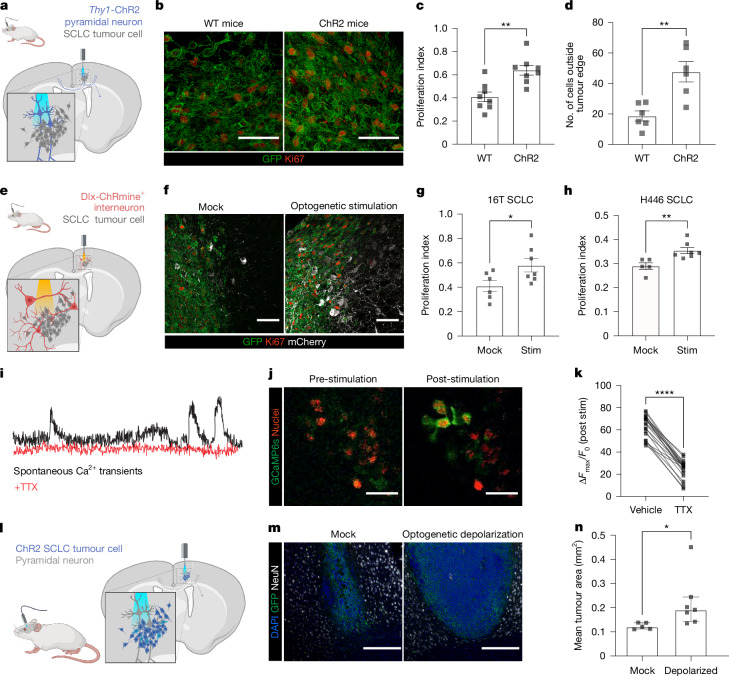
Neuronal circuit activity and downstream tumour membrane depolarization drive tumour progression. **a**, Paradigm for in vivo optogenetic stimulation of *Thy1*-ChR2 (ChR2) pyramidal premotor cortical projection neurons in awake behaving mouse with SCLC tumour allografted into the M2 cortex. **b**, Representative immunofluorescence of mouse 16T SCLC brain tumours (GFP) allografted in the cortex of wild-type (WT) or optogenetically stimulated ChR2 mice. Proliferating cells are labelled with Ki67. Scale bars, 50 µm. **c**, Quantification of data in **b** (*n* = 8 wild-type and *n* = 8 ChR2 mice, *P* = 0.0016). **d**, Quantification of SCLC cells (per 500 µm) invading beyond the tumour edge following optogenetic stimulation of wild-type or ChR2 mice (*n* = 6 wild-type and *n* = 6 ChR2 mice, *P* = 0.0029). **e**, Paradigm for in vivo optogenetic stimulation of Dlx-ChRmine (red-light-sensitive channelrhodopsin)-expressing GABAergic cortical interneurons in awake mice with SCLC tumours allografted into the M2 cortex. **f**, Representative immunofluorescence of mouse 16T SCLC brain tumours (GFP) allografted to the cortex of Dlx-ChRmine expressing mice (mCherry) following optogenetic stimulation (stim). Proliferating cells are labelled with Ki67. Scale bars, 50 µm. **g**, Quantification of data in **f** (*n* = 6 mock-stimulated mice and *n* = 7 stimulated mice, *P* = 0.0410). **h**, As in **f** for human H446 SCLC cells (*n* = 5 mock-stimulated and *n* = 7 stimulated mice, *P* = 0.0074). **i**, Two-photon in situ calcium imaging of GCaMP6s-expressing 16T SCLC cells in hippocampal allografts. Representative trace of spontaneous activity as measured by changes to GCaMP6s fluorescence in SCLC cells with (red) or without (black) administration of 0.5 µM TTX. **j**, Two-photon in situ calcium imaging of GCaMP6s-expressing SCLC cells in hippocampal allografts with Schaffer collateral stimulation (*n* = 6 slices, 4 mice). Representative frames shown before and after stimulation. Red denotes the tdTomato nuclear tag of SCLC cells; green denotes SCLC GCaMP6s. Scale bars, 25 µm. **k**, Quantification of GCaMP6s fluorescence in individual SCLC cells in response to electrical stimulation of CA1 Shaffer collateral axons with or without administration of 0.5 µM TTX (*n* = 22 cells, *P* < 0.0001). **l**, Paradigm for in vivo optogenetic depolarization of intracranial allografts of ChR2-expressing 16T SCLC cells. **m**, Representative immunofluorescence of ChR2-expressing SCLC allografts after mock or blue light-induced depolarization. Neuronal nuclei are labelled with NeuN and tumour cells are labelled with GFP. Scale bars, 200 µm. **n**, Quantification of mean tumour area from **m** (*n* = 5 mock and *n* = 7 depolarized mice, *P* = 0.0101). Data are mean ± s.e.m. (**c**,**d**,**g**,**h**); data are median ± interquartile range (**n**). Unpaired *t*-test (**c**,**d**,**g**,**h**); paired *t*-test (**k**); Mann–Whitney test (**n**). All tests are two-tailed. Drawings in **a**,**e**,**l** created in BioRender. Savchuk, S. (2025) https://BioRender.com/5fwotqm. Source data

**Extended Data Fig. 10:**
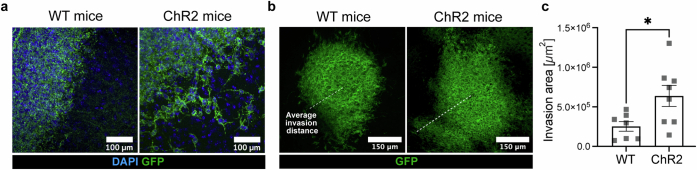
Neuronal circuit activity drives invasion of SCLC cells. **a**, Immunofluorescent images demonstrating cells expanding beyond the invasive edge of the tumor in SCLC brain allografts (GFP, green) of WT (left) or optogenetically stimulated ChR2 animals (right). Scale bar = 100 µm. **b**, Immunofluorescent images demonstrating increased tumor area in SCLC brain allografts of WT or ChR2 animals. Scale bar = 150 µm. **c**, Quantification of data in **b**, illustrating increased tumor spread from core after optogenetic stimulation (n = 7 WT, n = 8 ChR2 animals, p = 0.0267). Data are mean ± s.e.m for **c**. Analysis with two-tailed unpaired t-test for **c**. *P < 0.05. Source data

To examine the effects of neuronal activity on intracranial SCLC tumour proliferation, we employed in vivo optogenetic techniques in freely behaving mice. Here, mouse SCLC cells (16T) were allografted intracranially into the premotor cortex of mice expressing the blue light-sensitive opsin channelrhodopsin (ChR2) in Thy1+ deep layer cortical projection neurons or wild-type (non-ChR2-expressing) littermate controls (Fig. 4a). After a period of engraftment, optogenetic ferrules were placed over the premotor cortex and neurons were stimulated with blue light. Successful stimulation of the cortical circuit in ChR2-expressing mice was verified by the observance of a complex motor behavioural output, circular ambulation. Assessed histologically 24 h after optogenetic stimulation of cortical neuronal activity, 16T SCLC cells exhibited a robust increase in proliferation rate (approximately 40% Ki67^+^ SCLC cells in mock-stimulated wild-type mice, approximately 60% Ki67^+^ SCLC cells in optogenetically stimulated ChR2-expressing mice; Fig. 4b,c). Further, there was a clear increase in the spread of SCLC cells into the brain, with more cells migrating outside of the edge of the tumour mass into the normal brain parenchyma in mice with optogenetically stimulated neuronal activity (Fig. 4d and Extended Data Fig. 10a–c). Together, these results indicate that cortical neuronal activity can promote the progression of intracranial SCLC growth.

GABAergic synaptic neurotransmission comprised the majority of the signalling detected in electrophysiological studies, and was in fact depolarizing in nature. Thus, after observing that cortical circuit stimulation drives SCLC proliferation, we sought to determine the distinct contribution of GABAergic interneurons. We grafted mice with GFP-expressing SCLC (16T or H446) cells, together with an adeno-associated viral vector to genetically express ChRmine, a red-shifted channelrhodopsin^48^, in Dlx-expressing GABAergic interneurons in the mouse cortex (Fig. 4e). The mouse cortex was then optogenetically stimulated in awake, behaving mice to induce GABAergic interneuron activity. Control mice were identically manipulated with mock stimulation. After 24 h, SCLC proliferation was assessed histologically using Ki67 to measure proliferation index. We found that in vivo optogenetic stimulation of GABAergic interneurons promoted proliferation of both 16T and H446 allografted SCLC cells (Fig. 4f–h) recapitulating the increased proliferation as a result of increased motor circuit activity and demonstrating a stark tumour-promoting role of GABAergic neuronal signalling in intracranial SCLC growth.

## SCLC membrane depolarization regulates growth

**Extended Data Fig. 11:**
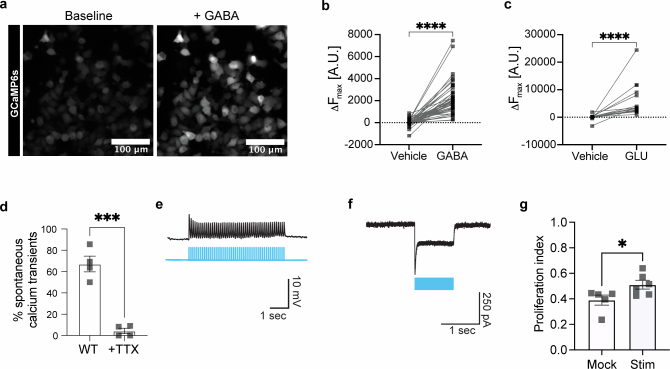
Membrane depolarization drives SCLC pathogenesis. **a**, Representative calcium imaging of GCaMP6s-expressing 16T SCLC cells at baseline (left) and after application of 1 mM GABA. Scale bar = 100 μm. **b**, Quantification of GCaMP6s fluorescence in individual SCLC cells (from **a**) in response to administration of 1 mM GABA (n = 44 cells, p < 0.0001). **c**, As in **b**, but with administration of 1 mM glutamate (n = 19 cells, p < 0.0001). **d**, Quantification of the percent of cells exhibiting spontaneous calcium transients as depicted in Fig. 4i in SCLC tumors at baseline or with the addition of 0.5 µM TTX (p = 0.0002). **e**, Whole-cell patch-clamp voltage trace from ChR2-expressing SCLC cells (16T-ChR2) in response to blue light-induced depolarization. The lower panel represents the timing of blue light delivery. **f**, as in **e**, but demonstrating whole-cell patch-clamp current trace from ChR2-expressing SCLC cells (16T-ChR2). **g**, Quantification of proliferation index of 16T-ChR2 SCLC cells in mock-stimulated tumors or membrane depolarized tumors (n = 5 mock, n = 6 depolarized mice, p = 0.0460). Data are plotted as mean ± s.e.m. for **d, g**. Analysis with two-tailed paired t-test for **b-c**, two-tailed unpaired t-test for **d, g**. ****P < 0.0001, ***P < 0.001, **P < 0.01, *P < 0.05. Source data

To define the role of SCLC membrane depolarization occurring as a result of synaptic neurotransmission, we visualized the neurotransmitter-mediated currents in SCLC cells using calcium imaging. After applying either glutamate or GABA individually to 16T SCLC cells engineered to express the genetically encoded calcium indicator GCaMP6s, both neurotransmitters elicited clear calcium transients in these malignant cells (Extended Data Fig. 11a–c), further evidence that these neurotransmitters induce depolarization. We then performed in situ two-photon calcium imaging of GCaMP6s-expressing 16T SCLC cells allografted into the hippocampus. Similar to observations using electrophysiology, both spontaneous and axonal stimulation-evoked calcium transients in the SCLC cells were observed (Fig. 4i,j). These transients were blocked by the addition of TTX, indicating their dependence on neuronal activity (Fig. 4k and Extended Data Fig. 11d).

Given the depolarizing SCLC currents and consequent calcium transients described above, we hypothesized that membrane depolarization itself provides a functional benefit to SCLC tumours in the brain. We used optogenetics to directly depolarize 16T SCLC cells engineered to express ChR2. We confirmed functionality of the ChR2 construct in SCLC cells with patch clamp electrophysiology (Extended Data Fig. 11e,f). These cells were allografted into the mouse cortex (Fig. 4l). After a period of engraftment, blue light was delivered via a fibre optic placed at the surface of the brain to directly depolarize these ChR2-expressing SCLC tumour cells in vivo. Control mice were identically manipulated with mock stimulation. Membrane depolarization resulted in a robust growth effect in SCLC, with an increased SCLC proliferation index (Extended Data Fig. 11g) and the overall size of the tumour approximately doubling after three optogenetic depolarization sessions compared with identically manipulated, mock-stimulated control mice (Fig. 4m,n). Together, these studies illustrate that SCLC cells in the brain utilize neuronal activity and consequent neurotransmitter-mediated membrane depolarization to fuel progression of the tumour.

## Reciprocal neuron–SCLC interactions

**Fig. 5:**
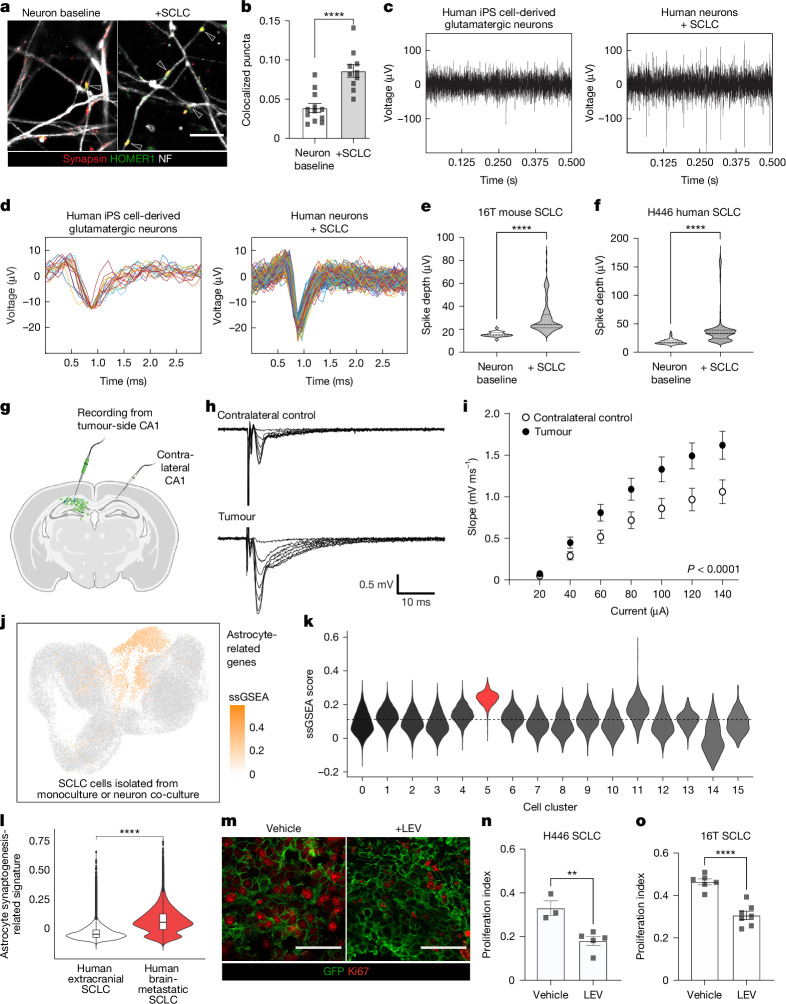
SCLC induces neuronal hyperexcitability. **a**, Immunofluorescence of human iPS cell-derived neurons in monoculture (left) or co-cultured with 16T SCLC cells (right). Arrowheads indicate colocalized pre-synaptic (synapsin) and post-synaptic (HOMER1) puncta along neuronal processes (neurofilament (NF)). Scale bar, 25 µm. **b**, Quantification of data in **a** (per 10 µm neurofilament length, *n* = 12 coverslips for neuron baseline and *n* = 10 coverslips for co-culture, *P* < 0.0001). **c**, Representative 500 ms recording of human iPS cell-derived glutamatergic neurons at baseline versus co-cultured with 16T SCLC cells using MEA (*n* = 6 per condition). **d**, Representative traces of spike amplitude in human iPS cell-derived glutamatergic neurons at baseline versus co-cultured with 16T SCLC cells. **e**, Quantification of data in **c**,**d** (*n* = 24 spikes in neuron baseline and *n* = 168 spikes in co-culture condition, *P* < 0.0001). **f**, As in **e**, but for human H446 SCLC cells (*n* = 363 spikes in neuron baseline and *n* = 2,721 spikes in co-culture, *P* < 0.0001). **g**, Paradigm for local field recordings in SCLC hippocampal allografts. Created in BioRender. Savchuk, S. (2025) https://BioRender.com/5fwotqm. **h**, Representative traces of local field potential in response to local stimulation of tumour allografts and control contralateral hippocampus. **i**, Extracellular local field potential (fEPSP) slope in response to various axonal stimulation intensities in the tumour-infiltrated or control contralateral hippocampus (data fit to a nonlinear regression and compared using the extra-sum-of-squares *F*-test; *n* = 24 tumour and *n* = 25 control across 6 mice, *P* < 0.0001). **j**, GSEA of scRNA-seq data from 16T SCLC cells isolated from either monoculture or neuron co-culture (Fig. 2f,g) reveals a distinct cluster that is enriched for astrocyte-related genes. All gene lists in Supplementary Table 1. **k**, Quantification of ssGSEA scores for astrocyte-related genes across the 16 identified clusters detects upregulation of astrocyte signature in cluster 5 (highlighted in red). Statistical testing in Supplementary Table 2. **l**, Expression of astrocyte synaptogenesis-related genes in cells isolated from patient lung primary, recurrent or non-brain-metastatic lesions^45^ (*n* = 16) versus patient SCLC brain metastases (*n* = 12, *P* < 0.0001). **m**, Representative immunofluorescence of human H446 SCLC (GFP) xenografted in cortex of mice treated with vehicle or levetiracetam (LEV). Proliferating cells are labelled with Ki67. Scale bars, 50 µm. **n**, Quantification of data in **m** (*n* = 3 vehicle and *n* = 5 levetiracetam-treated mice, *P* = 0.0065). **o**, As in **n**, but for mouse 16T SCLC cells (*n* = 6 vehicle and n = 7 levetiracetam-treated mice, *P* < 0.0001). Data are mean ± s.e.m. (**b**,**i**,**n**,**o**); violin plot (**e**,**f**,**k**); and violin and box plot (**l**). Unpaired *t*-test (**b**,**n**,**o**); Mann–Whitney test (**e**,**f**); one-way ANOVA with Tukey correction (**k**); pairwise Wilcoxon rank sum test (**l**). All tests are two-tailed. Source data

**Extended Data Fig. 12:**
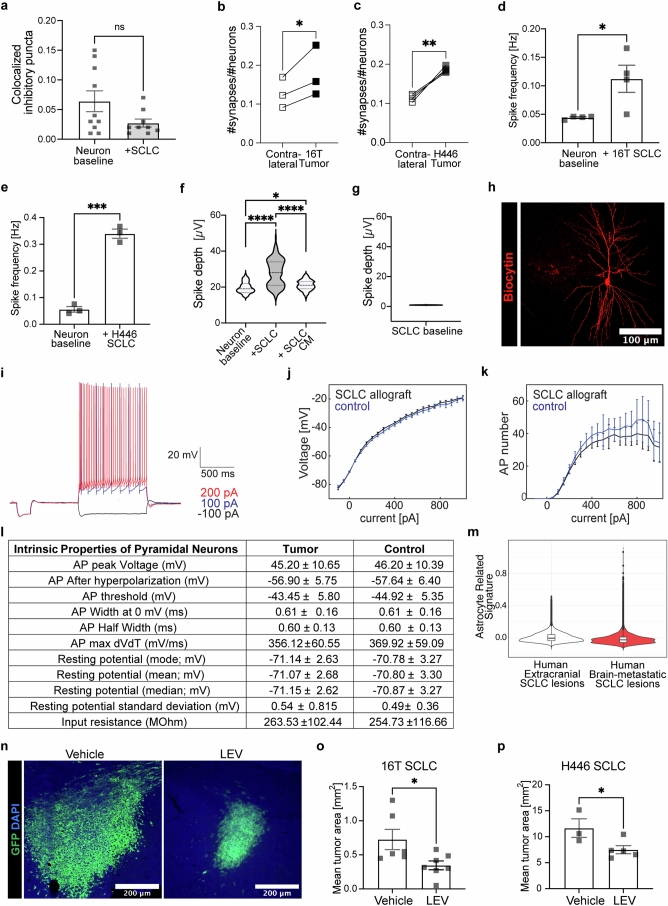
Bidirectional neuron-SCLC interactions drive peritumoral hyperexcitability. **a**, Quantification of the number of colocalized pre- and post-synaptic inhibitory puncta (quantified via colocalized synapsin-gephyrin staining) on neuronal processes at baseline vs. co-cultured with SCLC cells defined per 10 µm of neurofilament length (n = 10 coverslips in neuron baseline condition, n = 9 coverslips in SCLC co-culture condition). **b**, Quantification of the number of synapses per number of neurons within the region of murine 16T SCLC hippocampal allografts compared to contralateral normal hippocampi, demonstrating increased synaptic density in the tumor region compared to contralateral control hippocampus. Only regions of abundant (>5% cells) perisynaptic SCLC cells were considered (n = 3 mice, p = 0.0158). **c**, As in **b**, but for human H446 SCLC cells (n = 4 mice, p = 0.0037). **d**, Quantification of spike frequency in iPSC-derived glutamatergic neurons co-cultured with murine 16T SCLC cells (n = 4, p = 0.0296). **e**, as in **d**, but with the addition of human H446 SCLC cells (n = 3, p = 0.0005). **f**, Quantification of spike number and amplitude of iPSC-derived glutamatergic neurons at baseline or with the addition of conditioned media (CM) collected from SCLC cells (n = 91 spikes at baseline, n = 430 spikes in co-culture, n = 236 spikes in CM-treated condition, p < 0.0001). CM-treated neurons exhibit some elevation in spike depth but not to the degree of those recorded from neurons grown in direct co-culture with SCLC cells. **g**, Quantification of spike number and amplitude of SCLC cultured alone. **h**, Biocytin (red)-filled pyramidal neuron in the area of the tumor cells in situ. Scale bar = 100 μm. **i**, Representative current clamp currents and induced action potentials measured in pyramidal neurons in response to varying current injections (−100 pA, black; 100 pA, blue; 200 pA, red). **j**, Current to voltage relationship of action potentials in neurons from either the allograft (tumor-bearing) hippocampus or control contralateral hippocampus (n = 65 SCLC-associated, 32 control neurons). **k**, Current to action potential firing frequency relationship in neurons from either the allograft (tumor-bearing) hippocampus or control contralateral hippocampus (n = 65 SCLC-associated, 32 control neurons). **l**, Table listing cell-intrinsic properties of pyramidal neurons from either the allograft (tumor-bearing) hippocampus or control contralateral hippocampus (n = 65 SCLC-associated, 32 control neurons). **m**, Expression of astrocyte-related gene signature (Supplementary Table 1) taken from scRNAseq analysis of cells isolated from patient lung primary, recurrent or non-brain-metastatic lesions (n = 16, data made publicly available by Chan et al. 2021) vs. cells from patient SCLC brain metastases (n = 12). **n**, Representative immunohistochemistry imaging of overall tumor burden in animals allografted with 16 T SCLC cells (green) and treated with vehicle or levetiracetam (LEV). Scale bar = 200 μm. **o**, Quantification of data in **n** (n = 6 vehicle, n = 7 levetiracetam, p = 0.0309). **p**, As in **o**, but for animals xenografted with human SCLC-N subtype H446 (n = 3 vehicle, n = 5 levetiracetam, p = 0.0472). Data are mean ± s.e.m for **a, d, e, o, p**, violin plot for **f, g**, violin and box blot for **m** (box defined by 25^th^ percentile, median, and 75^th^ percentile, whiskers extend to min/max, dots represent outliers). Analysis with two-tailed unpaired t-test for **a, d, e, o, p**, two-tailed paired t-test for **b, c**, Kruskal-Wallis test for **f**. ****P < 0.0001, ***P < 0.001, **P < 0.01, *P < 0.05. Source data

As cancers metastatic to brain are often associated with seizures^49,50^, we next assessed whether SCLC cells in the brain could reciprocally affect neurons in the microenvironment. We first quantified the colocalization of pre-synaptic puncta (synapsin) with post-synaptic puncta (HOMER1) on neurons in our SCLC-neuron co-cultures. We found a marked increase in the number of synapses between glutamatergic neurons co-cultured with 16T SCLC cells compared with neurons in monoculture (Fig. 5a,b). No change in gephyrin-labelled synaptic puncta between GABAergic interneurons was detected (Extended Data Fig. 12a). Similarly, in our in vivo electron micrographs of allografted or xenografted tissue, we found an increased number of neuron-to-neuron synapses in regions of the tumour compared with the non-tumour-bearing contralateral controls (Extended Data Fig. 12b,c). Testing the functional contribution of SCLC cells to neuronal hyperexcitability using multielectrode array (MEA) electrophysiology, we found that neurons exhibited increased activity in the presence of SCLC cells (either 16T or H446), measured by increased spike number, amplitude and frequency (Fig. 5c–f and Extended Data Fig. 12d,e). Minimal increase in spike amplitude was observed when conditioned medium from SCLC cells was applied instead of direct co-culture (Extended Data Fig. 12f). We recorded no spikes in SCLC monoculture (Extended Data Fig. 12g).

We then assessed these findings in vivo by performing local field recordings of allografted hippocampal tissue (Fig. 5g). Compared with the contralateral non-tumour-infiltrated hippocampus, we found increased field potentials in regions of the tumour (Fig. 5h,i). We next sought to determine whether this effect was due to any cell-intrinsic changes in pyramidal neurons associated with SCLC and performed in situ whole-cell recordings of pyramidal neurons either integrated in regions of the tumour or in the contralateral (control, non-tumour-infiltrated) hippocampus. Between the tumour-infiltrated and control hippocampi, no distinct differences in neuronal properties were noted (Extended Data Fig. 12h–l).

Astrocytes are known to have a central role in the form and function of neuronal circuits and astrocyte-like glioma cells have been known to create a hyperexcitable neuronal microenvironment^51–53^. As described above, ultrastructural examination of allografted tissue revealed not only post-synaptic positioning of SCLC cells, but also perisynaptic associations of SCLC cells with neighbouring neurons (Extended Data Fig. 6b–d), reminiscent of a normal astrocyte position in tripartite synapses^54^. Querying our scRNA-seq data of SCLC cells in response to neuronal exposure, we found that neuronal co-culture increased the expression of a gene signature associated with an astrocytic phenotype^55–57^ in a distinct subpopulation of cells (cluster 5; Supplementary Tables 1 and 2), not overlapping with the population defined by an upregulated synaptic signature (Fig. 5j,k). In our single-cell dataset comparing human SCLC brain metastases to extracranial sites, we saw no enrichment in the astrocytic signature in the brain (Extended Data Fig. 12m). These findings may in part be driven by an already existing astroglial-like SCLC population that has previously been described within the lung^58^. We therefore evaluated a synaptogenesis-promoting astrocytic gene signature and found an upregulation within separate metaprogrammes of patient brain-metastatic SCLC cells (Fig. 5l). Together, these observations suggest that SCLC can remodel and reinforce neuron–SCLC interactions in the brain.

## Therapeutically targeting neuron–SCLC interactions

Given the increased neuronal activity in the region of the tumour and the idea that glutamatergic and GABAergic synaptic signalling to the tumour drives SCLC growth within the brain, we sought to harness neuron–SCLC interactions for treatment using existing clinically available drugs. We utilized levetiracetam, a commonly used anti-seizure drug that acts by interfering with synaptic vesicle release, to determine the therapeutic potential of reducing activity-mediated signalling in SCLC. After a 2-week treatment regimen of daily levetiracetam treatment (20 mg kg^−1^), both 16T and H446 SCLC-bearing mice demonstrated a significant reduction in malignant cell proliferation (Fig. 5m–o) and tumour burden (Extended Data Fig. 12n–p) in the brain compared with vehicle treated controls. These findings suggest that disrupting neuron–SCLC interactions will be a central component of effectively treating SCLC.

## Discussion

The influence of neuronal activity on SCLC pathophysiology demonstrated here adds to the growing evidence highlighting the importance of neurobiology in cancer. These findings indicate that SCLC cells that have colonized the brain take advantage of neuronal activity through both paracrine and synaptic mechanisms to enhance growth and invasion, while reciprocally increasing neuronal excitability and activity. Developing a longitudinal understanding of the roles of these neural pathways across tumour initiation, growth and metastases will be critical to developing novel therapies that target nervous system interactions with SCLC. Further, emerging evidence demonstrates that melanoma and breast cancer cells that have colonized the brain rely on glutamatergic synaptic input for growth^59^, extending these findings to other types of brain metastases and emphasizing the need to target mechanisms of activity-dependent growth in other non-glial cancers.

In the future, further investigation of the specific molecular signalling cascades that mediate this activity-driven progression of SCLC may reveal therapeutic vulnerabilities. Other mechanisms of activity-mediated growth, including the identity of the paracrine factors that promote SCLC proliferation remain to be defined. This study identifies glutamatergic and GABAergic synaptic signalling in SCLC tumours growing in the brain, but dose-dependent effects and potential roles of other neurotransmitters and neuropeptides have not yet been explored. The growth-promoting effect of SCLC membrane depolarization—also evident in glioma^2,16^—warrants further investigation of the voltage-sensitive mechanisms of cancer cell proliferation. The stark effect of denervation on pulmonary SCLC pathobiology raises questions about the distinct involvement and relative contributions of the different axonal subpopulations within the vagus nerve, the answers to which would provide potential therapeutic targets such as specific neurotransmitter receptors and inform potential translation of these findings to the bedside. Further, evaluating the possible effects of neuronal activity on other cells in the lung tumour microenvironment, including immune, vascular and other stromal cells may reveal additional indirect effects of nervous system activity on SCLC pathogenesis. SCLC is a disease with limited treatment options and poor prognosis. Thus, disrupting the functional interactions between neurons and SCLC in lung and in brain represents a promising therapeutic strategy for this lethal cancer.

## Methods

### Mice and housing conditions

All in vivo experiments were conducted in accordance with protocols approved by the Brigham and Women’s Hospital Institutional Animal Care and Use Committee (IACUC) and Stanford University IACUC. Mice were housed according to the standard guidelines with free access to food and water in a 12 h light:12 h dark cycle.

For brain tumour allograft experiments, NSG mice (NOD-SCID-IL2R gamma chain-deficient, the Jackson Laboratory) were used. Male and female mice were used equally. According to the IACUC guidelines, signs of morbidity rather than maximal tumour volume was used as indication for termination of brain allograft mouse experiments. Mice were euthanized if they exhibited signs of neurological disease or if they lost 15% or more of their body weight. For in vivo optogenetic stimulation of the premotor circuit (M2), *Thy1*-ChR2; NSG or WT; NSG mice were used.

For lung tumour experiments, mice were euthanized when they exhibited signs of sickness behaviour (such as dyspnea, abnormal gait or posturing, or ill-groomed fur) or lost >15% of body weight in accordance with IACUC guidelines. No limits were exceeded in any mouse experiments. In these experiments, *Rb1*^*fl/fl*^;*Trp53*^*fl/fl*^;*p130*^*fl/fl*^, luciferase-expressing (RPR2-luc) genetic mouse models were used as described previously^39,44^. In these mice, lung tumours and later distant metastases form spontaneously after intratracheal administration of Adeno-CMV-Cre (University of Iowa Vector Core) at 2 months of age as described^44^ and following a published protocol^60^. To study MYC-driven lung tumour models, we used RPM mice^42^. These mice were infected intratracheally as above with Adeno-CMV-Cre or Adeno-CGRP-Cre at 3 to 4 months of age.

### Intracranial allografts

All SCLC brain allografts were performed as described^2^. In brief, a single-cell suspension from cells cultured from either 16T-mGFP or NCI-H446-GFP SCLC neurospheres was prepared in sterile HBSS immediately before surgery. Mice at postnatal day (P)21–35 were anaesthetized with 1–4% isoflurane and placed in a stereotactic apparatus. The cranium was exposed via midline incision under aseptic conditions. 70,000 cells in 3 µl sterile HBSS were stereotactically injected into the M2 region of the cortex through a 31-gauge burr hole using a digital pump at an infusion rate of 0.4 µl min^−1^ and a 31-gauge Hamilton syringe. Stereotactic coordinates for *Thy1*-ChR2 mouse allografts used were as follows: 0.5 mm lateral to midline, 1.0 mm anterior to bregma, −1.5 mm deep to cortical surface. At the completion of infusion, the syringe needle was allowed to remain in place for a minimum of 2 min, then withdrawn at a rate of 0.875 mm min^−1^ to minimize backflow of the injected cell suspension. To generate mice with interneuron-specific expression of light-sensitive ion channels, 1 µl of AAV8-Dlx-ChRmine-p2A-mCherry^61^ (virus titre = 6.9 × 10^12^) (a gift from K. Deisseroth) was unilaterally injected using a Hamilton Neurosyringe and 450 Stoelting stereotaxic injector over 5 min. These mice then received allografts of 16T-mGFP or xenografts of NCI-H446-GFP SCLC cells as described above at 0.8 mm lateral to midline, 1.0 mm anterior to bregma, −1.2 mm deep relative to the cortical surface. Allografts of 16T-mGFP-ChR2 SCLC cells were performed following the procedure as above with exception for the following alterations: 15,000 cells were injected in 1 µl sterile HBSS 0.8 mm lateral to midline, 1.0 mm anterior to bregma, −1.2 mm deep relative to cortical surface. For electrophysiology experiments and electron microscopy tissue analysis, 16T-mGFP cells were allografted into the CA1 region of the hippocampus at the following coordinates: 1.5 mm lateral to midline, 1.8 mm posterior to bregma, −1.35 mm deep to the cortical surface.

### In vivo optogenetic manipulation

For in vivo optogenetic stimulation of M2 region of *Thy1*-ChR2; NSG or WT; NSG mice, a single stimulation paradigm was employed as previously described^1^. In brief, a fibre optic ferule was placed 1 week following or simultaneously with and ipsilateral to the SCLC allografts. After 1–2 weeks to allow for recovery from the procedure, the mice were connected to a 100-mW 473-nm diode-pumped solid-state laser system with a mono fibre patch cord, which freely permits wakeful behaviour of the mice. Pulses of light with approximately 4 mW measured output at tip of the patch cord were administered at a frequency of 20 Hz for periods of 30 s, followed by 90 s recovery periods, for a total session duration of 30 min. The mice were euthanized after 24 h post stimulation, and brains were collected for histological analysis. For stimulation of cortical interneurons in Dlx-ChRmine mice, 595-nm light was used at 40 Hz frequency and 10 ms width. Pulses of light with approximately 10 mW measured output at tip of the patch cord were administered.

For in vivo optogenetic depolarization of SCLC cells, ChR2–YFP (pLV-ef1-ChR2(H134R)-eYFP WPRE) construct (generated by the laboratory of K. Deisseroth and placed in the piggyback transposon system by M. Su in the laboratory of M.M.) was lentivirally transduced into 16T SCLC cells, which were then allografted into premotor cortex (M2) following the procedure described above. A fibre optic ferule was implanted during the same surgery ipsilateral to the cell injection site at following coordinates: 0.8 mm lateral to midline, 1.0 mm anterior to bregma, −0.9 mm deep relative to cortical surface. At 1 week post-allograft and for three consecutive days, all mice were connected to the laser system to receive blue light or mock stimulations at a frequency of 10 Hz for periods of 30 s, followed by 90 s recovery periods for a total session duration of 30 min. Mice were euthanized 24 h after the final (3rd) stimulation session.

### Immunohistochemistry of patient tissue

Patient tissue samples were obtained with informed consent and analysed in accordance with institutional review board-approved protocols. Immunohistochemistry of patient SCLC brain metastases tissue samples was performed on formalin-fixed paraffin-embedded tissue sections per standard protocols including deparaffinization, antigen retrieval, incubation with primary antibody and detection per the manufacturers’ instructions. The following antibodies were used: mouse anti-Ki67 (Dako/Agilent), mouse anti-neurofilament (Ventana Roche; prediluted). Staining for Ki67 was performed on a Leica Bond III automated stainer. Staining for neurofilament was performed on a Ventana Ultra automated stainer. Proliferation index was determined by quantifying the fraction of Ki67^+^ cells out of the total number of cells in the region.

### Single-cell sequencing from SCLC-neuron co-culture

16T SCLC cells were cultured alone or in neuron co-cultures with or without 1 µM TTX for 24 h per protocol below and collected in PBS 0.5% BSA, 1 mM EDTA (Invitrogen), 1x DNAse (Worthington Biochemical). GFP-negative cells were collected in parallel to serve as negative control during FACS. Calcein violet (Thermo Fisher) was used to label live cells. GFP^+^calcein^+^ cells were sorted and collected then lysed and combined into droplets with barcoded beads which captured the mRNA then used for reverse transcription with the The Chromium Single Cell Gene Expression platform (10X Genomics) per the manufacturer’s instructions. We then followed the rest of the 10X standard or high-throughput protocols and used the Dual Index Kit TT Set A for library production. The experiment was repeated for a total of three biological replicates.

Processing of fastq files from monoculture and co-culture samples was performed individually using the 10X Genomics Cell Ranger 7.1.0 based on the mm10 mouse genome reference, with the incorporation of the eGFP sequence. Seurat^62^ (v.5.0.1) was employed for data loading at the individual sample level. Subsequently, the scCB2^63^ (v.1.12.0) package was utilized to filter out empty droplets, employing an FDR threshold of 0.01 to identify real cells, while potential doublets were removed using the scDblFinder^64^ (v.1.16.0) package. Cells with no GFP expression, exhibiting a high fraction of mitochondrial molecules (>5%) and those expressing a low number of unique genes (indicating low library complexity) were excluded.

Samples from the same replicate were merged, and highly variable genes were selected using the Seurat package. The relative expression values of these highly variable genes were used for principal component analysis (PCA). The number of PCA components for each replicate was determined based on achieving a cumulative proportion greater than 80% in the PCA plot. Subsequently, UMAP embeddings were generated and cells were clustered using Seurat’s Louvain algorithm-based FindClusters function.

Differentially expressed genes were identified using the SeuratWrapper’s (v.0.3.19) RunPrestoAll function. Genes detected in a minimum of 30% of the cells within each cluster, with at least a 0.25-fold mean log difference, were subjected to statistical testing using the Wilcoxon rank sum test with Bonferroni correction for multiple testing. Genes with adjusted *P* value  <  0.05 were retained.

GSEA was conducted using the fgsea^65^ (v.1.28.0) and genekitr^66^ (v.1.2.5) packages, exploring GO, KEGG, REACTOME, Hallmarks, Biocarta and WikiPathways databases. Finally, the GSVA^67^ (v.1.50.0) package was used to calculate the ssGSEA scores for synaptome, astrocytes, and cell proliferation signatures.

### Single-cell sequencing from human primary or metastatic SCLC lesions

#### Human SCLC brain tissue transcriptomic library preparation

Frozen tissues were processed as described^68,69^. Tissue blocks were embedded in optimal cutting temperature (Tissue-Tek, Sankura 4583), and then sectioned on a Leica CM1950 cryostat (Leica) into 20-µm-thick curls (generating up to 20 curls for each sample). These were then placed in 5-ml tubes (Eppendorf), washed with ice-cold PBS (Thermo Fisher Scientific, 10010023), centrifuged at 400*g* for 2 min, and the supernatant was discarded. The tissue was resuspended in 1 ml Salt Tris (ST) buffer (146 mM NaCl, 10 mM Tris-HCL pH 7.5, 1 mM CaCl_2_ and 21 mM MgCl2 in ultrapure water) with 0.03% Tween-20 (Sigma Aldrich, P7949; TST buffer), supplemented with 0.1% BSA (New England Biolabs, B9000S) and optionally 40 U ml^−1^ RNAse inhibitor (RNAse OUT, Thermo Fisher Scientific). The suspension was mechanically dissociated by pipetting 15 times with a 1-ml pipette and incubated on ice for 5 min. Afterward, the pipetting step was repeated, and the reaction was quenched with 4 ml ST buffer, with or without RNAse inhibitor. The mixture was filtered through pre-wetted 70-µm nylon mesh filters (Thermo Fisher Scientific) into 50-ml conical tubes, washed with 5 ml ST buffer, and centrifuged at 500*g* for 5 min to isolate the nuclei. The nuclei pellet was resuspended in 100–400 µl ST buffer, filtered through a 40-µm mesh (Thermo Fisher Scientific), and counted using a Neubauer counting chamber (Bulldog Bio) after staining nuclear DNA with 50 µg ml^−1^ Hoechst 33342 (Thermo Fisher Scientific, H3570). Approximately 0.9 to 1.5 × 10³ nuclei were loaded into a Chromium Controller using ST buffer without RNAse inhibitor and processed with Chromium reagents and 5′V2 capture kits (1000006 and 1000263) from 10X Genomics. Following reverse transcription and cleanup, cDNA libraries were prepared per manufacturer protocols, including one additional cycle of amplification to account for the lower RNA content in nuclei compared with whole cells. Final sequencing libraries were created using the library construction kit (1000190) and Dual Index Kit TT Set A (1000215) and sequenced on an Illumina NovaSeq S4 platform with 2× 150 bp paired-end reads, achieving a minimum of 25,000 reads per cell.

#### Filtering background noise in gene expression matrices

Demultiplex FASTQ files from raw RNA-sequencing reads were aligned using CellRanger v.6.1.1 (10X Genomics) to the GRCh38 genome^70^. Gene counts were quantified with CellRanger’s ‘count’ function, including intronic reads. The feature_bc_matrix.h5 files generated by CellRanger were used as inputs for the ‘remove-background’ function in CellBender v.0.2.0, which removed ambient RNA gene counts and empty droplets^71^. The CellRanger metric ‘expected-cells’ defined the ‘Expected Number of Cells’ parameter, and the total-droplets-included parameter was set to a value between 10,000 and 40,000, chosen from the plateau region of the barcode-rank plot generated by CellRanger.

#### Quality control and normalization

Each generated matrix for was processed with R v.4.1.1 and Seurat v.4.1.0 on a per-sample basis^72^. Filters were applied based on the Seurat pipeline to retain only cells with 500–10,000 detected genes, 1,000–60,000 unique molecular identifiers and less than 10% mitochondrial gene content. Scrublet v.0.2.1 was used to identify and remove doublets, with the expected doublet rate set between 2.5% and 7.5%, depending on the initial loading rate^73^. Following Seurat’s pipeline, the data were log-normalized using the NormalizeData function. The top 2,000 variable genes from each sample were identified with the FindVariableFeatures function, and the resulting matrix was centred and scaled with the ScaleData function. All signatures were computed through entered the gene list and a merged Seurat object of all samples on a per-cohort basis using the AddModuleScore function provided by Seurat.

#### Integration of cohort samples

Individual Seurat samples were integrated using Seurat canonical correlation analysis (CCA) pipeline to remove batch effects from individual samples. Samples from the labelled Chan cohort from and CUIMC cohort were integrated separately^72^. Per the CCA pipeline, SelectIntegrationFeatures and FindIntegrationAnchors was then run to select 2,000 anchors between each sample with the top 50 dimensions from CCA to define search space for integration, using the raw RNA counts assay for each sample. IntegrateData was then run using the previously defined anchors to generate the integrated dataset. The integrated data were then scaled, and clustered using ‘FindNeighbors’ with 10 dimensions and FindClusters using a resolution of 0.5. UMAPs were calculated with the top 30 PCA dimensions, using Seurat’s RunUMAP.

#### Cell-type identification

Cell types were initially labelled using SingleR v.1.8.0 using the built in BlueprintEncodeData reference^74^. Immune cells identified through this process were used as a diploid reference for inferCNV v.1.10.1 to infer chromosomal copy number alteration (CNA) profiles for each cell. A minimum average read count threshold of 0.1 per gene was applied for reference nuclei. The ‘subcluster’ setting was used for clustering, and results were denoised with the default ‘sd_amplifier’ value of 1.5. InferCNV used a hidden Markov model to predict CNA levels, and the proportion of scaled CNAs was averaged across all chromosomes for each cell^74^. Malignant cells were identified using sample-specific thresholds based on these average values, which distinguished immune and non-immune CNA levels.

Non-malignant cell types were further analysed following CCA-based integration. Clustering of non-malignant cells was performed with the FindClusters function at varying resolutions, followed by differential gene expression analysis with FindAllMarkers^72^. Broad cell-type annotations were assigned manually based on established marker genes identified as differentially expressed in each cluster.

#### Non-negative matrix factorization

Non-negative matrix factorization (NMF) was employed for feature extraction and dimensionality reduction on non-negative gene expression data^75^. The NMF function implemented in RcppML v.0.3.7, was selected for its computational efficiency by minimizing reconstruction error and optimizations matrix factorization^76^. RcppML operates directly on raw count matrices and incorporates L1 regularization with reproducible factor scaling, enabling robust handling of ambiguous zeroes in single-cell data. NMF iteratively decomposing the data matrix into two lower-dimensional non-negative matrices, a basis matrix representing gene programmes and a coefficient matrix capturing the effect of each gene programmes on each cell. To incorporate prior biological knowledge, the supervised framework from Tagore et. al was adapted^77^. NMF was run on each sample to identify latent gene programmes, followed by rank-factor. Genes were pre-filtered to retain the top 7,000 genes based on total counts from the filtered RNA assay, serving as input.

Metaprogrammes (also referred to as consensus factors) were identified through co-correlation analysis using Spearman’s correlation applied to sample-specific factors generated by NMF. The optimal number of consensus factors was determined using silhouette and distortion scores. Ward’s clustering was then performed on individual factors, with the optimal number of consensus factors setting the clustering thresholds to define metaprogrammes^77^. This analysis was conducted on a per-cohort basis.

#### Metaprogramme correlation with annotated hallmarks of tumour heterogeneity

To annotate the biological functions underlying each metaprogramme, gene signatures were defined based on the top genes with the highest normalized contributions to each metaprogramme. Functional characterization of these gene signatures was performed through GSEA using recently described transcriptional hallmarks of tumour heterogeneity^46^. To assess the similarity between gene signatures, the Jaccard similarity index and a derived correlation metric were calculated. The Jaccard similarity index quantifies overlap between two sets as the size of their intersection divided by the size of their union. To facilitate interpretation in a heatmap, pairwise Jaccard similarity scores were scaled linearly to a correlation-like metric ranging from 0 to 1. The resulting heatmap highlights pairwise relationships between gene signatures, with metaprogrammes exhibiting the highest correlation with a given metaprogramme annotated as representing biologically related functions.

#### Normalized gene contribution towards metaprogrammes

For each metaprogramme, genes were ranked based on a weighted Stouffer-integrated expression value. The top 50 genes per metaprogramme, determined by this ranking, were selected for downstream analyses. Within each sample, cell barcodes from the H matrix generated by NMF were assigned to a specific metaprogramme based on their highest association with a corresponding factor. These assignments linked individual barcodes to metaprogrammes according to their factor membership. To evaluate the gene contributions to each metaprogramme, an expectation-maximization Gaussian mixture model (EM-GMM) was applied to the normalized gene expression matrices^77^. This model assessed the modality of gene expression distributions, enabling the identification of peaks that correspond to the ‘normalized gene contribution’ for each metaprogramme. These contributions were subsequently scaled between 0 and 1 across all metaprogrammes to ensure comparability.

#### GSEA across metaprogrammes

The top 50 ranked genes for each metaprogramme, based on the weighted Stouffer-integrated expression values, were used for functional enrichment analysis. GSEA was performed using EnrichR v.3.1, leveraging the Hallmarks of MSigDB v.7.4.1 and GO Biological Process 2021 databases^78^. Enrichment results were aggregated across cohorts, with the top-ranked gene sets identified for each metaprogramme. The *P* values and *q* scores returned by EnrichR were scaled and visualized to highlight significant functional associations for each metaprogramme.

### Sample preparation and image acquisition for electron microscopy

NSG mice were engrafted with either 16T-mGFP or NCI-H446-GFP cells into mouse hippocampi. Three weeks after engraftment, mice were sacrificed by transcardial perfusion with Karnovsky’s fixative: 2% glutaraldehyde (EMS 16000) and 4% paraformaldehyde (PFA) (EMS 15700) in 0.1 M sodium cacodylate (EMS 12300), pH 7.4. The samples were then post-fixed in 1% osmium tetroxide (EMS 19100) for 1 h at room temperature, washed 3 times with ultrafiltered water, then en bloc stained for 2 h at room temperature. Samples were dehydrated in graded ethanol (50%, 75% and 95%) for 15 min each at 4 °C; the samples were then allowed to equilibrate to room temperature and were rinsed in 100% ethanol 2 times, followed by acetonitrile for 15 min. Samples were infiltrated with EMbed-812 resin (EMS 14120) mixed 1:1 with acetonitrile for 2 h followed by 2:1 EMbed-812:acetonitrile for 2 h. The samples were then placed into EMbed-812 for 2 h, then placed into TAAB capsules filled with fresh resin, which were then placed into a 65 °C oven overnight. Sections were taken between 40 and 60 nm on a Leica Ultracut S (Leica) and mounted on 100-mesh Ni grids (EMS FCF100-Ni). For immunohistochemistry, microetching was done with 10% periodic acid and eluting of osmium with 10% sodium metaperiodate for 15 min at room temperature on parafilm. Grids were rinsed with water three times in between and followed by 0.5 M Glycine quench. Grids were incubated in blocking solution (0.5% BSA, 0.5% Ovalbumin in PBST) at room temperature for 20 min. Primary rabbit anti-GFP (1:300; MBL International) was diluted in the same blocking solution and incubated overnight at 4 °C. The following day, grids were rinsed in PBS three times, and incubated in secondary antibody (1:10 10 nm Gold conjugated IgG TED Pella 15732) for 1 h at room temperature and rinsed with PBST followed by water. For each staining set, samples that did not contain any GFP-expressing cells were stained simultaneously to control for any non-specific binding. Grids were contrast stained for 30 s in 3.5% uranyl acetate in 50% acetone followed by staining in 0.2% lead citrate for 90 s. Samples were imaged in the tumour mass within the CA1 region of the hippocampus or within the contralateral normal hippocampus using a JEOL JEM-1400 transmission electron microscope at 120 kV and images were collected using a Gatan Orius digital camera.

### Electron microscopy data analysis

Sections from the allografted hippocampi of mice were imaged as above using transmission electron microscopy. Here, 36 sections of 16T-mGFP across 6 mice were analysed. Electron microscopy images were taken at 6,000× with a field of view of 15.75 μm^2^. Synapses were inspected by two individual investigators. SCLC cells were counted and analysed after unequivocal identification of immunogold particle labelling with four or more particles. For identification of synaptic structures, all three of the following criteria had to be clearly met: (1) visually apparent synaptic cleft; (2) presence of synaptic vesicle clusters in a cell on one side of the cleft; and (3) identification of clear post-synaptic density on the cell on opposite side of cleft. For identification of neuron-to-tumour synapses, the post-synaptic cell had to exhibit clear immunogold particle labelling. Tumour cells in perisynaptic positions were identified when an immunogold-particle-positive SCLC cell was seen apposed to or surrounding the synaptic structures between two other immunogold-particle-negative cells. Density of both types of synaptic relationships (neuron-to-tumour synapses, neuron-to-tumour perisynaptic connections) were quantified as the number of connections per number of SCLC cells identified within the region.

### Histology

Mice with intracranial tumour allografts were anaesthetized with intraperitoneal avertin (tribromoethanol), then transcardially perfused with 20 ml of ice-cold PBS. Brains were fixed in 4% PFA overnight at 4 °C, then cryoprotected in 30% sucrose, embedded in Tissue-Tek O.C.T. (Sakura) and sectioned in the coronal plane at 40 μm using a sliding microtome. Lung tumour mice were perfused as above but also with 10 ml ice-cold 4% PFA. The lungs were inflated with 1–3 ml 2% UltraPure Low Melting Point Agarose (Invitrogen). Lungs and livers were fixed overnight at 4 °C on a shaker, then transferred to 70% ethanol and sectioned at 15 μm for H&E and immunohistochemistry, or cryoprotected in 30% sucrose and sectioned at 150 μm on a cryotome for visualizing tumour innervation.

For immunofluorescence staining, the coronal sections were incubated in blocking solution (3% normal donkey serum, 0.3% Triton X-100 in TBS) at room temperature for 45 min, followed by an overnight incubation with primary antibodies in antibody diluent solution (1% normal donkey serum in 0.3% Triton X-100 in TBS) at 4 °C. On the next day, after a 5-min rinse in TBS, sections were incubated with DAPI (1 μg ml^−1^ in TBS, Thermo Fisher) for 5 min, and rinsed again with TBS for 5 min. Afterwards, slices were incubated in secondary antibody solution at 4 °C overnight, then washed thrice in TBS and mounted with ProLong Gold Mounting medium (Life Technologies). All images were acquired with Zen 3.4 and analysed using Fiji ImageJ 2.1.0. For quantifying SCLC proliferation, average number of Ki67^+^ SCLC cells divided by total number of SCLC cells labelled by GFP was calculated within regions of interest either in axon-rich/axon-poor areas of the tumour or in response to various optogenetic manipulations. For quantification of SCLC spread, average distance from the core was measured as longest distance from the initial site of injection to outer core of the tumour. For quantification of tumour invasion, average number of SCLC cells that migrated out of the circumscribed tumour edge over ~500 μm was calculated.

For visualizing lung and liver tumour innervation with immunofluorescence, 150-μm-thick sections were processed as above, except primary antibody incubation time was extended to 72 h. Streptavidin Alexa Fluor 594 conjugate (Invitrogen) was used to visualized airway epithelium^79^. To quantify lung and liver tumour burden, five equidistant H&E sections from each organ were evaluated by a pathologist blinded to the experimental conditions to estimate percent of section area occupied by the tumour. Any section with <5% of tissue estimated to be occupied by tumour was given a score of 0, 5–25% was given a score of 1, 25–50% was given a score of 2, 50–75% was given a score of 3, and >75% was given a score of 4. The score for each mouse was then generated as an average across the five sections.

The following primary antibodies were used: chicken anti-GFP (Aves Labs, 1:500), rabbit anti-MAP2 (EMD Millipore, 1:500), mouse anti-NeuN (EMD Millipore, 1:500), rabbit anti-Ki67 (Abcam, 1:500), guinea pig anti-synapsin (Synaptic Systems, 1:500), rabbit anti-HOMER1 (Synaptic Systems, 1:500), mouse anti-neurofilament (Abcam, 1:500), mouse anti-nestin (Abcam, 1:1,000), guinea pig anti-VChAT (Synaptic Systems, 1:200), mouse anti-TH (Abcam, 1:200), and rat anti-MBP (Abcam, 1:200). The following secondary antibodies were used (all Jackson Immuno Research, 1:500): DyLight 405 Donkey Anti-Mouse IgG, Alexa Fluor 488 Donkey Anti-Chicken IgG, Alexa Fluor 488 Donkey Anti-Guinea Pig IgG, Alexa Fluor 488 Donkey Anti-Rabbit IgG, Alexa Fluor 594 Donkey Anti-Rabbit IgG, Alexa Fluor 647 Donkey Anti-Mouse IgG, Alexa Fluor 647 Donkey Anti-Rabbit IgG, Alexa Fluor 647 Donkey Anti-Rat IgG.

### Cell culture

The mouse 16T SCLC line was derived from a primary tumour from the lungs of an Rb/p53 mutant mouse^44^. 16T-GFP cells were generated by transducing 16T cells with pLV-CMV-GFP followed by FACS selection^28^. These cells are grown as neurospheres (unless otherwise stated) in 10% FBS medium consisting of DMEM (Invitrogen) and 1× liquid antibiotic-antimycotic (Invitrogen). The spheres were dissociated using TrypLE (Gibco) for seeding of in vitro experiments. For human cell lines, SCLC22H, H69, CORL47, and H526 were generously provided by M. Oser. SCLC22H was cultured in DMEM plus 10% FBS (Cytiva) and 1× Glutamax. H69, CORL47, H446 and H526 were cultured in RPMI with 10% FBS and 1× Glutamax. H1048 was cultured in RPMI plus 10% FBS, 1× Glutamax, and 1× Insulin transferrin selenium (Fisher Scientific). Primary small airway epithelial cells (HSAECs) were purchased from ATCC and grown in airway epithelial cell basal media supplemented with bronchial epithelial cell growth components (ATCC). All cultures were monitored by short tandem repeat fingerprinting for authenticity throughout the culture period and mycoplasma testing was routinely performed.

### Co-culture of SCLC cells with primary mouse neurons

Neurons were isolated from the brains of CD1 mice using the Neural Tissue Dissociation Kit - Postnatal Neurons (Miltenyi), followed by the Neuron Isolation Kit, Mouse (Miltenyi) per the manufacturer’s instructions. After isolation, 300,000 neurons were plated onto circular glass coverslips (Electron Microscopy Services) pre-treated for 20 min at 37 °C with poly-l-lysine (Sigma) and then 3 h at 37 °C with 5 μg ml^−1^ mouse laminin (Thermo Fisher). Neurons were cultured in BrainPhys neuronal medium (Stemcell Technologies) supplemented with 1× Glutamax (Invitrogen), pen/strep (Invitrogen), B27 supplement (Invitrogen), BDNF (10 ng ml^−1^; Shenandoah), and GDNF (5 ng ml^−1^; Shenandoah), TRO19622 (5 μM; Tocris), β-mercaptoethanol (1×, Gibco) and 2% fetal bovine serum. Half of the medium was replenished on day in vitro (DIV) 1 and 5-fluoro-2'-deoxyuridine (UFDU) was added at 1 μM. This was repeated at DIV 3. On DIV 5, half of the medium was replaced with serum-free medium in the morning. In the afternoon, the medium was again replaced with half serum-free medium containing 75,000 SCLC cells dissociated from neurospheres or attached cultures with TrypLE. Tumour cells were cultured with neurons for 24 h and then fixed with 4% PFA for 20 min at room temperature and stained for immunofluorescence analysis as described below.

### Co-culture of SCLC cells with human iPS cell-derived glutamatergic neurons

iPS cell lines were obtained from the Brigham and Women’s Hospital NeuroHub Core Facility and all permissions were received for use (from BWH NeuroHub Core and Rush Alzheimer’s Disease Center’s Biospecimen Distribution Committee). Induced neurons were generated from BR33 iPS cells as described^80^. In brief, iPS cells were plated in mTeSR1 medium at a density of 95,000 cells per cm^2^ on Matrigel-coated plates for viral transduction. Viral plasmids were obtained from Addgene (plasmids #19780, #52047 and #30130). FUdeltaGW-rtTA was a gift from K. Hochedlinger (Addgene plasmid #19780; http://n2t.net/addgene:19780; RRID: Addgene_19780). TetO-FUW-EGFP was a gift from M. Wernig (Addgene plasmid #30130; http://n2t.net/addgene:30130; RRID: Addgene_30130). pTet-O-Ngn2-puro was a gift from M. Wernig (Addgene plasmid #52047; http://n2t.net/addgene:52047; RRID: Addgene_52047). Lentiviruses were obtained from Alstem with ultrahigh titres (~1 × 10^9^) and used at the following concentrations: pTet-ONGN2-puro: 0.13 µl, 50,000 cells; TetO-FUW-eGFP: 0.13 µl, 50,000 cells; FUdelta GW-rtTA: 0.13 µl, 50,000 cells. Transduced cells were dissociated with 3:1 DPBS: Accutase (Stemcell Technologies) + ROCKi (10 µM, Stemcell Technologies) and plated onto Matrigel-coated plates at 200,000 cells per cm^2^ in StemFlex medium + ROCKi (10 µM, Stemcell Technologies) (day 0). On day 1, medium was changed to KSR medium with doxycycline (2 µg ml^−1^, Sigma). Doxycycline was maintained in the medium for the remainder of the differentiation. On day 2, medium was changed to 1:1 KSR: N2B medium with puromycin (5 µg ml^−1^, GIBCO). Puromycin was maintained in the medium throughout the differentiation. On day 3, medium was changed to N2B medium + 1:100 B27 supplement (Life Technologies). From day 4 on, cells were cultured in NBM medium + 1:50 B27 + puromycin (5 µg ml^−1^) + BDNF, GDNF, CNTF (10 ng ml^−1^, Peprotech). Half medium changes were performed every 2–3 days. Around D10, puromycin was removed from medium. Around D12-D-15, SCLC cells were added at a ratio of 1:3 (cancer cells:neurons). Cultures were monitored over the next ten days in the case of MEA recordings. For histological analyses, SCLC cells were added in the presence of 1 μM TTX (Tocris), 50 μM MK801 (Selleck Chemicals), 50 μM CNQX (Tocris), or vehicle. Co-cultures were then fixed and analysed 24 h later in the case of proliferation assays, and 5 days after co-culture in the case of synapse quantification.

### Co-culture of SCLC cells with human iPS cell-derived GABAergic neurons

Induced pluripotent stem cell lines were obtained from the Brigham and Women’s Hospital NeuroHub Core Facility and all permissions were received for use (from BWH NeuroHub Core and Rush Alzheimer’s Disease Center’s Biospecimen Distribution Committee). In brief, iPS cells were plated in mTeSR1 medium at a density of 95,000 cells per cm^2^ on Matrigel-coated plates for viral transduction. Viral plasmids were obtained from Addgene (plasmids #19780, #97329 and #97330). FUdeltaGW-rtTA was a gift from K. Hochedlinger (Addgene plasmid #19780). TetO-Ascl1-puro was a gift from M. Wernig (Addgene plasmid #97329; http://n2t.net/addgene:97329; RRID: Addgene_97329). DLX2-hygro was a gift from M. Wernig (Addgene plasmid #97330; http://n2t.net/addgene:97330; RRID: Addgene_97330). Lentiviruses were obtained from Alstem with ultrahigh titres (~1 × 10^9^) and used at the following concentrations: TetO-Ascl1-T2A-Puro: 0.13 µl, 50,000 cells; DLX2-hygro: 0.13 µl, 50,000 cells: FUdelta GW-rtTA: 0.13 µl, 50,000 cells. Transduced cells were dissociated with 3:1 DPBS: Accutase + ROCKi (10 µM) and plated onto Matrigel-coated plates at 200,000 cells per cm^2^ in StemFlex medium + ROCKi (10 µM) (day 0). On day 1, medium was changed to N2B medium with doxycycline (2 µg ml^−1^) and forskolin (10 µM, Sigma). Doxycycline and forskolin were maintained in the medium for the remainder of the differentiation. On day 2, medium was replaced with fresh N2B medium. On day 3, medium was changed to N2B medium + puromycin (10 µg ml^−1^, GIBCO). On day 4 onwards, cells were cultured in N2B medium + puromycin (8 µg ml^−1^). From day 5 onwards, cells were cultured with NBM medium + 1:50 B27 + puromycin (5 µg ml^−1^) + BDNF, GDNF, CNTF (10 ng ml^−1^) + doxycycline (2 µg ml^−1^) + forskolin (10 µM) + AraC (2 µM, Sigma). Half medium changes were performed every 2–3 days. Around day 14, puromycin, doxycycline, AraC, and forskolin were removed from medium. Around day 20 to day 25, SCLC cells were added at a ratio of 1:3 (cancer cells:neurons). For histological analyses, SCLC cells were added in the presence of 1 μM TTX (Tocris), 20 μM gabazine (Tocris) or vehicle. Co-cultures were then fixed and analysed 24 h later in the case of proliferation assays, and 5 days after co-culture in the case of synapse quantification.

### Induced neuron protocol medium

KSR medium: Knockout DMEM, 15% KOSR, 1× MEM-NEAA, 55 µM β-mercaptoethanol, 1× Glutamax (Life Technologies). N2B medium: DMEM/F12, 1× GlutaMAX (Life Technologies), 1× N2 supplement B (StemCell Technologies), 0.3% dextrose (d-(+)-glucose, Sigma). NBM medium: neurobasal medium, 0.5× MEM-NEAA, 1× GlutaMAX (Life Technologies), 0.3% dextrose (d-(+)-glucose, Sigma).

### Conditioned media assays

For conditioned media assays, either glutamatergic or GABAergic iPS cell-derived neurons were cultured as above. On day 17–18, neurons were replenished with a full medium change with or without the presence of 1 μM TTX. Conditioned medium was collected 24 h later for immediate use. Mouse 16T cells were then plated (30,000 cells in a 48 well plate) with 500 μL neuronal conditioned medium (again in the absence or presence of TTX) with the addition of 10 μM EdU to each well. After 24 h, cells were fixed cells with 4% PFA and stained using Click-iT 594 EdU kit and protocol (Invitrogen). All conditioned media assays were performed alongside direct co-culture assays described above.

### EdU incorporation assay

EdU staining was performed on glass coverslips in 24-well plates which were precoated with poly-l-lysine (Sigma) and 5 μg ml^−1^ mouse laminin (Thermo Fisher). Neurosphere cultures were dissociated with TrypLE and plated onto coated slides with 10 μM of EdU. After 24 h the cells were fixed with 4% PFA in PBS for 20 min and then stained using the Click-iT 594 EdU kit and protocol (Invitrogen) with or without additional antibody staining and mounted using Prolong Gold mounting medium (Life Technologies). Proliferation index was determined by quantifying the fraction of EdU-labelled cells/GFP-labelled cells using confocal microscopy at 40× magnification.

### Synaptic puncta staining and visualization

For immunohistochemistry, fixed coverslips were incubated in blocking solution (3% normal donkey serum, 0.3% Triton X-100 in TBS) at room temperature for 1 h. Primary antibodies guinea pig anti-synapsin1/2 (1:500; Synaptic Systems), rabbit anti-HOMER1 (1:500; Synaptic Systems), rabbit anti-gephyrin (1:300, Cell Signaling Technologies), or mouse anti-neurofilament (1:500; Abcam) in 0.3% Triton X-100 in TBS and incubated overnight at 4 °C. Samples were then rinsed 3 times in TBS and incubated in secondary antibody solution (Alexa 488 donkey anti-guinea pig IgG; Alexa 594 donkey anti-rabbit IgG, and Alexa 647 donkey anti-mouse IgG, all at 1:500 (Jackson Immuno Research)) in antibody diluent solution at 4 °C overnight. Coverslips were rinsed three times in TBS and mounted with ProLong Gold Mounting medium (Life Technologies). Images were collected using a 63× oil-immersion objective on a Zeiss LSM800 confocal microscope and processed with Airyscan. Colocalization of puncta was quantified as described^2^.

### MEA recordings

All MEA recordings were taken and analysed using the Axion Biosystems platform. Prior to culturing, 6-well Axion plates were coated with poly-l-lysine and laminin. Day 4 iNs (created as described^80^) were then thawed and plated in neurobasal medium, at 100,000 cells per well. For the initial week prior to co-culture, iNs were subjected to a half medium change every 3–4 days. Doxycycline and puromycin treatment were stopped after day 10 to allow for the addition of tumour cells. On day 14–15, SCLC cells were added at 30,000 cells per well MEA plates. MEA plates were recorded every day for 10 min for up to 2 weeks after co-culture. All spike numbers and amplitude were assessed using proprietary Axion Software.

### Electrophysiology

For all electrophysiology experiments, 35,000 16T-GFP cells were allografted into the CA1 hippocampus of 4–6 week old NSG mice. Three weeks after allograft, brain slices were obtained using standard techniques. Mice were anaesthetized by isoflurane inhalation and perfused transcardially with ice-cold ACSF containing (in mM) 125 NaCl, 2.5 KCl, 25 NaHCO_3_, 2 CaCl_2_, 1 MgCl_2_, 1.25 NaH_2_PO_4_ and 25 glucose (295 mOsm kg^−1^). Brains were blocked and transferred into a slicing chamber containing ice-cold ACSF. Coronal slices of hippocampus were cut at 300-μm thickness with a Leica VT1000s vibratome in ice-cold ACSF, transferred for 10 min to a holding chamber containing choline-based solution consisting of (in mM) 110 choline chloride, 25 NaHCO_3_, 2.5 KCl, 7 MgCl_2_, 0.5 CaCl_2_, 1.25 NaH_2_PO_4_, 25 glucose, 11.6 ascorbic acid, and 3.1 pyruvic acid at 34 °C then transferred to a secondary holding chamber containing ACSF at 34 °C for 30 min and subsequently maintained at room temperature (20–22 °C) until use. All recordings were obtained within 5 h of slicing. Both choline solution and ACSF were constantly bubbled with 95% O_2_/5% CO_2_.

Individual brain slices were transferred into a recording chamber, mounted on an upright microscope (Olympus BX51WI or Scientifica SliceScope Pro 1000) and continuously superfused (2–3 ml min^−1^) with ACSF and bubbled with 95% O_2_/5% CO_2_ warmed to 32–34 °C by passing it through a feedback-controlled in-line heater (SH-27B; Warner Instruments). Cells were visualized through 40× or 60× water immersion objectives with either infrared differential interference contrast optics or epifluorescence to identify GFP^+^ cells. For whole-cell voltage clamp recording, patch pipettes (2–4 MΩ) pulled from borosilicate glass (Sutter Instruments) were filled with internal solution containing (in mM) 135 CsMeSO_3_, 10 HEPES, 1 EGTA, 3.3 QX-314 (Cl^−^ salt), 4 Mg-ATP, 0.3 Na-GTP, 8 sodium phosphocreatine (pH 7.3 adjusted with CsOH; 295 mOsm·kg−1). sEPSCs and spontaneous inhibitory post-synaptic currents (sIPSCs) of GFP^+^ cells were recorded for 5 min at –70 or 0 mV holding potential. For membrane current variance, 10 consecutive sweeps, each 10 s long, recorded in voltage clamp at −70 mV holding potential were analysed by calculating the s.d. of the current after applying a 3-point median filter. To record evoked excitatory post-synaptic currents (eEPSCs) and evoked inhibitory post-synaptic currents (eIPSCs), the membrane voltages were clamped at –70 mV or 0 mV. For perforated-patch recording eIPSCs (gramicidin D, 40–60 μg ml^−1^) with access resistance 40–60 MΩ, variable holding membrane voltages were applied to generate an *I*–*V* curve and identify chloride’s equilibrium potential. Extracellular stimulation was performed with a stimulus isolation unit (MicroProbes, ISO-Flex), bipolar electrodes (75 μm apart, PlasticOne or MicroProbes) which were placed away 100–400 μm along the CA1–CA2 axis from the recorded cells. Stimuli were delivered at 10 s intervals with 0.1 ms duration and 100–200 μA amplitude. In some recordings, to confirm whether sEPSCs or eIPSCs were glutamatergic or GABAergic, 10 μM NBQX and CPP (Sigma) or 10 μM gabazine (Tocris) were added to bath ACSF. For perforated patched recordings, 10 μM NBQX and CPP were in bath continuously. Pyramidal cells in allografted and contralateral sites of hippocampus were recorded in whole-cell current clamp to study intrinsic properties. Patch pipettes were filled with internal solution containing (in mM) 135 potassium methanesulfonate, 10 HEPES, 1 EGTA, 4 Mg-ATP, 0.4 Na-GTP, 8 sodium phosphocreatine (pH 7.3 adjusted with KOH; 295 mOsm kg^−1^). From a resting potential of −70 mV, currents were injected for 1,000 ms at 50 pA steps from −100 to 1,000 pA. Intrinsic properties of hippocampus pyramidal cells were analysed with software in Matlab.

Extracellular local field potentials (fEPSPs) were recorded from hippocampal allograft and contralateral sites under current clamp (*I* = 0 mode). A bipolar electrode (75 μm spacing, MicroProbes) was placed in stratum radiatum near the allograft or in the correspondent contralateral site. Recording glass electrodes (2–3 MΩ) filled ACSF were place 100–200 μm perpendicularly away from the stimulating bipolar electrode at a depth of 50 100 μm. The stimulation currents were delivered at 20 s intervals with 0.1 ms duration and variable intensities from 20 μA to 140 μA using 20 μA steps and repeated 10 times. The average slopes of fEPSPs for each current intensity were measured.

The resting membrane potential of SCLC was assessed through cell-attached configuration (Rseal > 1 GΩ) using the procedure previously described in Antel et al.^81^. Glass pipettes were filled with a solution containing (in mM) 145 KCL, 2 MgCl2, 5 HEPES (pH was adjusted to 7.3 with KOH). A voltage protocol consisting of hold at 0 mV, stepping to 100 mV and ramping back to −120 mV over 137.5 ms was applied to GFP^+^ cells. Five to ten consecutive traces were recorded and averaged. All data were acquired with a MultiClamp 700B amplifier (Molecular Devices) and digitized at 3 kHz with a National Instruments data acquisition device (NI USB- 6343).

### Calcium imaging

For calcium imaging, the genetically encoded calcium indicator GCaMP6s was lentivirally transduced into mouse SCLC 16T (pLV-ef1-GCaMP6s-P2A-nls-tdTomato). In this case, SCLC cells containing the GCaMP6s reporter can be identified using the tdTomato nuclear tag. These cells were isolated and grafted into the CA1 region of the hippocampus as described above. Two-photon calcium imaging experiments were performed using Prairie Ultima XY upright two-photon microscope for tissue slices equipped with an Olympus LUM Plan FI W/IR-2 40× water immersion objective. The temperature of the perfusion medium, ACSF as described above, was kept at 30 °C, and perfused through the system at rate of 2 ml min^−1^. Excitation light was provided at a wavelength of 920 nm through a tunable Ti:Sapphire laser (Spectra Physics Mai Tai DeepSee) to allow for excitation of both tdTomato and GCaMP6s. The actual laser power reaching the scanhead for each scope is dynamically controlled by Pockels cells via software interface. Pockels cell were set at 10 for all experiments, and photomultiplier tubes (PMTs) were set at 800 for each channel. For these settings, power at back aperture of the objective was approximately 30 mW at 920 nm. The wavelength ranges for the emission filters were PMT1: 607 nm centre wavelength with 45 nm bandpass (full-width at half-maximum) and PMT2: 525-nm centre wavelength with 70-nm bandpass (full-width at half-maximum). Recordings were made at 0.65 frames per second (~1.5 Hz) for about 30 min in the case of spontaneous activity and 10 min in the case of response to periodic electrical stimulation. Cells were identified via the expression for the nuclear tdTomato tag and were only imaged in the area of interest, specifically in the CA1 region of the hippocampus. Similar to the electrophysiology paradigm, for neuronal stimulation experiments, the electrode was placed in the hippocampus to stimulate the neuronal inputs originating in CA3. For electrical stimulation, approximately 20 µA over 200 µs was delivered to local axons using a stimulating bipolar microelectrode. For inhibitor experiments, TTX was directly diluted into the ACSF perfusion medium at 500 nM, oxygenated, and delivered to the slices through the perfusion system.

### Calcium imaging analysis

Quantitative fluorescence intensity analysis was done on calcium transients that were reliably evoked by axonal stimulation. To determine the effect of TTX on the calcium transients in response to electrical stimulation of the CA3 Schaffer collaterals, the field of cells were stimulated three times in 1-min intervals to ensure synaptic connectivity. TTX (500 nM) was then perfused into the slices and the stim was repeated on the same field of cells to gauge direct effect of TTX on stimulation response. For analysis, regions of interest of each responding nucleus were set and Δ*F*_max_/*F*_0_ (maximum difference in fluorescence intensity normalized to background fluorescence) measurements were determined before and after TTX treatment.

### Visualization of human SCLC lung tumour gene expression

Expression data from 81 patients with SCLC primary tumours^38^ as FPKM values was accessed and plotted for genes of interest.

### Unilateral cervical vagotomy

Adult RPR2-luc mice (4–7 months old, 2–5 months from virus administration) weighing more than 17 g were anaesthetized with 1–4% isoflurane through a nose cone in a supine position. Skin of the ventral surface of the neck was shaved and aseptically prepared according to IACUC guidelines. Under a dissection microscope, a 1 cm midline skin incision was made, and salivary glands revealed were separated with blunt dissection to expose the airways. The right vagal nerve was then carefully dissected from the carotid sheath and cut at the cervical level posterior to the pharyngeal branch. Sham mice underwent the same surgical procedure for blunt dissection of the vagus nerve but the latter was left intact. The mice were monitored until recovery from anaesthesia for changes in heart rate and respiration and after that monitored bi-weekly for changes in weight, eating, drinking, and general activity. RPM mice were handled the same way, except vagotomy was performed within 1 week of virus administration.

### Bioluminescence imaging

For in vivo monitoring of tumour growth, bioluminescence imaging was performed using an IVIS imaging system (Xenogen). Mice were placed under 1–4% isofluorane anaesthesia and injected with luciferin substrate, then imaged in pronated position for intracranial allograft experiments or supine position for lung tumour mice. Baseline bioluminescence was used to randomize mice by a blinded investigator so that experimental groups contained an equivalent range of tumour sizes. For vagotomy experiments, each cohort of mice was imaged weekly for a total of 6 months.

### Survival studies

For survival studies, morbidity criteria used were either reduction of weight by 15% of initial weight, sickness behaviour (such as dyspnea, abnormal gait or posturing, or ill-groomed fur), or severe neurological motor deficits consistent with brainstem dysfunction (that is, hemiplegia or an incessant stereotyped circling behaviour seen with ventral midbrain dysfunction). Kaplan–Meier survival analysis using log-rank testing was performed to determine statistical significance.

### Mouse drug treatment studies

For all drug studies, NSG mice were xenografted as above with either 16T-GFP or NCI-H446-GFP cells and randomized to treatment group by a blinded investigator. One week post-engraftment, tumour-bearing mice were treated with systemic administration of levetiracetam (20 mg kg^−1^; Selleck Chemicals; formulated in a sterile saline solution) via intraperitoneal injection daily for 2 weeks. Controls were treated with an identical volume of vehicle.

### Statistical analyses

Statistical tests were conducted using Prism v.9.3.1 (GraphPad) software unless otherwise indicated. Gaussian distribution was confirmed by the Shapiro–Wilk normality test. For parametric data, unpaired two-tailed Student’s *t*-test or one-way ANOVA with Tukey’s post hoc tests to examine pairwise differences were used as indicated. Paired two-tailed Student’s *t*-tests were used in the case of same-cell or same-animal experiments (as in electrophysiological recordings and vagotomy experiments). For non-parametric data, a two-sided unpaired Mann–Whitney test was used as indicated, or a one-tailed Wilcoxon matched pairs signed rank test was used for same-cell experiments. Two-tailed log-rank analyses were used to analyse statistical significance of Kaplan–Meier survival curves. In all box-and-whiskers plots, whiskers indicate minimum and maximum values, the box extends to the 25th and 75th percentile, and the centre line is plotted at the median. In all violin plots, lines are drawn at the median and quartiles.

### Reporting summary

Further information on research design is available in the Nature Portfolio Reporting Summary linked to this article.

## Online content

Any methods, additional references, Nature Portfolio reporting summaries, source data, extended data, supplementary information, acknowledgements, peer review information; details of author contributions and competing interests; and statements of data and code availability are available at 10.1038/s41586-025-09492-z.

## Supplementary information

Reporting Summary

Supplementary Table 1 Gene expression signatures. Each tab labels the specific gene signatures queried in single-cell and single-nucleus datasets.

Supplementary Table 2 Statistical analysis for gene expression signatures. Statistical analyses provided for expression of astrocytic and synaptic gene signatures across distinct clusters in the mouse scRNA-seq dataset.

## Source data

Source Data Fig. 1

Source Data Fig. 2

Source Data Fig. 3

Source Data Fig. 4

Source Data Fig. 5

Source Data Extended Data Figs. 1–3, 6 and 10–12

## Data Availability

Sequencing data for SCLC cells isolated from neuronal co-cultures and human brain-metastatic SCLC are available at the Gene Expression Omnibus (GEO) under accessions GSE262422 and GSE303152, respectively. All other data are available in the manuscript or from the corresponding author upon reasonable request. Source data are provided with this paper.
