# Supplementary material for: Neuronal activity-dependent mechanisms of small cell lung cancer pathogenesis
Source: Nature. 2025 Sep 10;646(8087):1232–42. doi: 10.1038/s41586-025-09492-z (PMC12571889; doi:10.1038/s41586-025-09492-z)
Supplement: Supplementary file 1 — Reporting Summary [file 41586_2025_9492_MOESM1_ESM.pdf]

nature portfolio

Corresponding author(s): Humsa Venkatesh, Michelle Monje

Last updated by author(s): July 28, 2025

Reporting Summary

Nature Portfolio wishes to improve the reproducibility of the work that we publish. This form provides structure for consistency and transparency in reporting. For further information on Nature Portfolio policies, see our Editorial Policies and the Editorial Policy Checklist.

Statistics

For all statistical analyses, confirm that the following items are present in the figure legend, table legend, main text, or Methods section.

n/a	Confirmed	
☐	☒	The exact sample size (*n*) for each experimental group/condition, given as a discrete number and unit of measurement
☐	☒	A statement on whether measurements were taken from distinct samples or whether the same sample was measured repeatedly
☐	☒	The statistical test(s) used AND whether they are one- or two-sided<br>*Only common tests should be described solely by name; describe more complex techniques in the Methods section.*
☒	☐	A description of all covariates tested
☐	☒	A description of any assumptions or corrections, such as tests of normality and adjustment for multiple comparisons
☐	☒	A full description of the statistical parameters including central tendency (e.g. means) or other basic estimates (e.g. regression coefficient) AND variation (e.g. standard deviation) or associated estimates of uncertainty (e.g. confidence intervals)
☐	☒	For null hypothesis testing, the test statistic (e.g. *F*, *t*, *r*) with confidence intervals, effect sizes, degrees of freedom and *P* value noted<br>*Give P values as exact values whenever suitable.*
☒	☐	For Bayesian analysis, information on the choice of priors and Markov chain Monte Carlo settings
☒	☐	For hierarchical and complex designs, identification of the appropriate level for tests and full reporting of outcomes
☒	☐	Estimates of effect sizes (e.g. Cohen's *d*, Pearson's *r*), indicating how they were calculated

Our web collection on statistics for biologists contains articles on many of the points above.

Software and code

Policy information about availability of computer code

Data collection	Confocal images were acquired using Zen 3.4 (blue edition). Electrophysiology data were collected using IgorPro v.5.05A. Ivis imaging was collected using Living Image 4.5.1.
Data analysis	Statisical tests was conducted using Graphpad Prism 9.3.1 for most analyses; Electrophysiology data were analyzed using Igor Pro v.5.05A software and MatLab (v6). Confocal microscopy image analysis was done using Fiji ImageJ 2.1.0 and Imaris 9.9.0. Calcium imaging data was analyzed using Fiji ImageJ 2.1.0. scRNAseq data was analyzed using Seurat (v5.0)

For manuscripts utilizing custom algorithms or software that are central to the research but not yet described in published literature, software must be made available to editors and reviewers. We strongly encourage code deposition in a community repository (e.g. GitHub). See the Nature Portfolio guidelines for submitting code & software for further information.

Data

Policy information about availability of data

All manuscripts must include a data availability statement. This statement should provide the following information, where applicable:

- Accession codes, unique identifiers, or web links for publicly available datasets
- A description of any restrictions on data availability
- For clinical datasets or third party data, please ensure that the statement adheres to our policy

Sequencing of SCLC cells isolated from neuronal co-cultures and of human brain metastatic SCLC are available on Gene Expression Omnibus (GEO) at GSE262422 and GSE303152, respectively. Processing of fastq files from scRNAseq data was performed using the 10x Genomics Cell Ranger 7.1.0 based on the mm10 mouse genome reference. Additional source data has been included in with manuscript.

Field-specific reporting

Please select the one below that is the best fit for your research. If you are not sure, read the appropriate sections before making your selection.

☒ Life sciences ☐ Behavioural & social sciences ☐ Ecological, evolutionary & environmental sciences

For a reference copy of the document with all sections, see nature.com/documents/nr-reporting-summary-flat.pdf

Life sciences study design

All studies must disclose on these points even when the disclosure is negative.

Sample size	Based on the variance of tumor growth in control mice, power calculations indicated use of at least 3-8 mice per condition to give 80% power to detect an effect size of 20% with a significance level of 0.05. For electrophysiological studies, all studies were replicated across multiple cohorts of mice to verify reproducibility. For all other experiments, a minimum of 3 biological replicates were performed.
Data exclusions	No data were excluded from the analyses.
Replication	For all in vitro/in vivo experiments, a minimum of at least 3 biological replicates were performed (listed in figure legends). For human data, replication was not possible.
Randomization	For IVIS imaging studies, baseline bioluminescence was used to randomize animals by a blinded investigator so that experimental groups contained an equivalent range of tumor sizes. For all other in vitro studies, all samples were randomized to different groups and treated and analyzed in the same way.
Blinding	For histological experiments, the analysis was done by blinded investigator. Electrophysiological studies were done in all tumor bearing mice and therefore could not be blinded.

Reporting for specific materials, systems and methods

We require information from authors about some types of materials, experimental systems and methods used in many studies. Here, indicate whether each material, system or method listed is relevant to your study. If you are not sure if a list item applies to your research, read the appropriate section before selecting a response.

Materials & experimental systems

n/a	Involved in the study	
☐	☒	Antibodies
☐	☒	Eukaryotic cell lines
☒	☐	Palaeontology and archaeology
☐	☒	Animals and other organisms
☐	☒	Human research participants
☒	☐	Clinical data
☒	☐	Dual use research of concern

Methods

n/a	Involved in the study	
☒	☐	ChIP-seq
☒	☐	Flow cytometry
☒	☐	MRI-based neuroimaging

Antibodies

Antibodies used	The following primary antibodies were used: chicken anti-GFP (Aves Labs, GFP-1020, Lot# GFP3717982, 1:500), rabbit anti-MAP2 (EMD Millipore, AB5622, Lot# 3826006, 1:500), mouse anti-NeuN (EMD Millipore, ab104225, 1:500), rabbit anti-Ki67 (Abcam, ab15580, Lot# GR3426431-1, 1:500), guinea pig anti-synapsin (Synaptic Systems, 106 308, Clone gp46.1,1:500), rabbit anti-homer1 (Synaptic Systems, 160 003, 1:500), rabbit anti-gephyrin (Cell Signaling, 14304S, Lot#1, 1:300), mouse anti-neurofilament (Abcam, ab7794, Clone NF-09, Lot: 1016126-1, 1:500), mouse anti-nestin (Abcam, ab6320, 1:1000), guinea pig anti-VAChT (Synaptic Systems, 139 105, Lot# 30021200, 1:200), rabbit anti-TH (EMD Millipore, AB152, Lot# 3114503, 1:200), rat anti-MBP (Abcam, ab7349, Clone#12, 1:200), Streptavidin, Alexa Fluor 594 conjugate (Invitrogen, S32356, Lot# 2387430, 1:200). For human tissue staining: anti-Ki-67 (Dako/Agilent, GA62661-2, Clone# MIB-1, 1:500), mouse anti-neurofilament (Ventana Roche, 760-2661, Clone# 2F11; prediluted).<br><br>The following secondary antibodies were used (all Jackson ImmunoResearch, 1:500): Alexa Fluor 488 Donkey Anti-Chicken IgG (703-545-155, 156558), Alexa Fluor 488 Donkey Anti-Guinea Pig IgG (706-545-148, 154921), Alexa Fluor 488 Donkey Anti-Rabbit IgG (711-545-152, 157934), Alexa Fluor 594 Donkey Anti-Rabbit IgG (711-585-152), Alexa Fluor 647 Donkey Anti-Chicken IgG (703-605-155, 157769), Alexa Fluor 647 Donkey Anti-Mouse IgG (715-605-150, 153251), Alexa Fluor 647 Donkey Anti-Rabbit IgG (711-605-152, 155990), Alexa Fluor 647 Donkey Anti-Rat IgG (712-605-153).
Validation	All antibodies have been validated in the literature and/or in Antibodypedia for use in mouse immunohistochemistry. To further validate the antibodies on our hands, we confirmed that each antibody stained in the expected cellular patterns and brain- and lung-wide distributions for immunohistochemistry.

Eukaryotic cell lines

Policy information about cell lines

Cell line source(s)	The murine 16T SCLC line was derived from individual primary tumors from the lungs of Rb/p53 DKO mice (created and supplied by Julien Sage). Human H446 SCLC line was originally purchased from ATCC. Patient-derived cultures of high-grade gliomas were generated from autopsy tissue (SU-DIPGVI, SU-DIPGXIIIFL).
Authentication	Sort Tandem Repeat (STR) fingerprinting is performed every 3 months on all cell cultures to ensure authenticity.
Mycoplasma contamination	All cell cultures are routinely tested for mycoplasma contamination and all cultures used tested negative.
Commonly misidentified lines (See ICLAC register)	No commonly misidentified lines were used.

Animals and other organisms

Policy information about studies involving animals; ARRIVE guidelines recommended for reporting animal research

Laboratory animals	Adult (aged 6-8 weeks) NSG mice (NOD-SCID-IL2R gamma chain-deficient, the Jackson Laboratory) or Thy1::ChR2;NSG mice were used for brain allografting. Adult Trp53flox/flox, Rb1flox/flox, p130flox/flox knockout (RPR2), luciferase (luc)-expressing SCLC genetic mice were used for vagotomy experiments. In all experiments, both male and female mice were used. Animals were housed in a facility where temperature was kept at 20-24C and humidity range was 35-65%.
Wild animals	No wild animals were used.
Field-collected samples	No field-collected samples were used.
Ethics oversight	Brigham and Women’s Hospital Institutional Animal Care and Use Committee (IACUC) and Stanford University IACUC.

Note that full information on the approval of the study protocol must also be provided in the manuscript.

Human research participants

Policy information about studies involving human research participants

Population characteristics	Patients that had undergone brain tumor resection and were diagnosed with SCLC were included in this study.
Recruitment	All samples were obtained from tissue archives. There was no prospective recruitment.
Ethics oversight	Columbia University Irving Medical Center Institutional Review Board

Note that full information on the approval of the study protocol must also be provided in the manuscript.
